# PANDA-view: an easy-to-use tool for statistical analysis and visualization of quantitative proteomics data

**DOI:** 10.1093/bioinformatics/bty408

**Published:** 2018-05-22

**Authors:** Cheng Chang, Kaikun Xu, Chaoping Guo, Jinxia Wang, Qi Yan, Jian Zhang, Fuchu He, Yunping Zhu

**Affiliations:** 1 State Key Laboratory of Proteomics, Beijing Proteome Research Center, Beijing Institute of Lifeomics, National Center for Protein Sciences (Beijing), Beijing, People’s Republic of China; 2 Beijing Key Laboratory of Human Computer Interactions, Institute of Software, Chinese Academy of Sciences, Beijing, People’s Republic of China; 3 Drug Research and Development Center, Shandong Drug and Food Vocational College, Weihai, People’s Republic of China

## Abstract

**Summary:**

Compared with the numerous software tools developed for identification and quantification of -omics data, there remains a lack of suitable tools for both downstream analysis and data visualization. To help researchers better understand the biological meanings in their -omics data, we present an easy-to-use tool, named PANDA-view, for both statistical analysis and visualization of quantitative proteomics data and other -omics data. PANDA-view contains various kinds of analysis methods such as normalization, missing value imputation, statistical tests, clustering and principal component analysis, as well as the most commonly-used data visualization methods including an interactive volcano plot. Additionally, it provides user-friendly interfaces for protein-peptide-spectrum representation of the quantitative proteomics data.

**Availability and implementation:**

PANDA-view is freely available at https://sourceforge.net/projects/panda-view/.

**Supplementary information:**

Supplementary data are available at *Bioinformatics* online.

## 1 Introduction

In the new era of life-omics, quantitative proteomics is becoming wide-spread with the rapid developments of high-resolution mass spectrometers (MS) and superior experiment strategies (Schubert *et al.*, 2017). Currently, there are lots of algorithms and tools for identification and quantification of -omics data. However, for most biological researchers who have few programming skills, the downstream analysis, such as the statistical analysis of differentially-expressed proteins (DEPs), remains a major challenge due to a lack of suitable and easy-to-use tools (Cappadona *et al.*, 2012). The few existing tools usually cannot perform both downstream analysis and data visualization with comprehensive methods. For example, GProX (Rigbolt *et al.*, 2011) and DanteR (Taverner *et al.*, 2012) did not provide necessary statistical tests and data visualization methods; Perseus (Tyanova *et al.*, 2016) and GiaPronto (Weiner *et al.*, 2017) included few normalization methods.

Here, to break the barrier between -omics data (especially the quantitative proteomics data) and the hidden biological/medical discoveries, we present an easy-to-use and light-weight tool, named PANDA-view, for statistical analysis and visualization of -omics data. PANDA-view can be compatible with other -omics tools by reading their results in comma-separated value (CSV) or tab-delimited text file format. It includes comprehensive methods for data normalization, imputation, DEP statistical test, unsupervised analysis and data visualization. Furthermore, it can provide a multi-level representation (from protein to MS spectrum) for the quantification results of PANDA (Chang *et al.*, 2018).

## 2 Methods

**Fig. 1.**
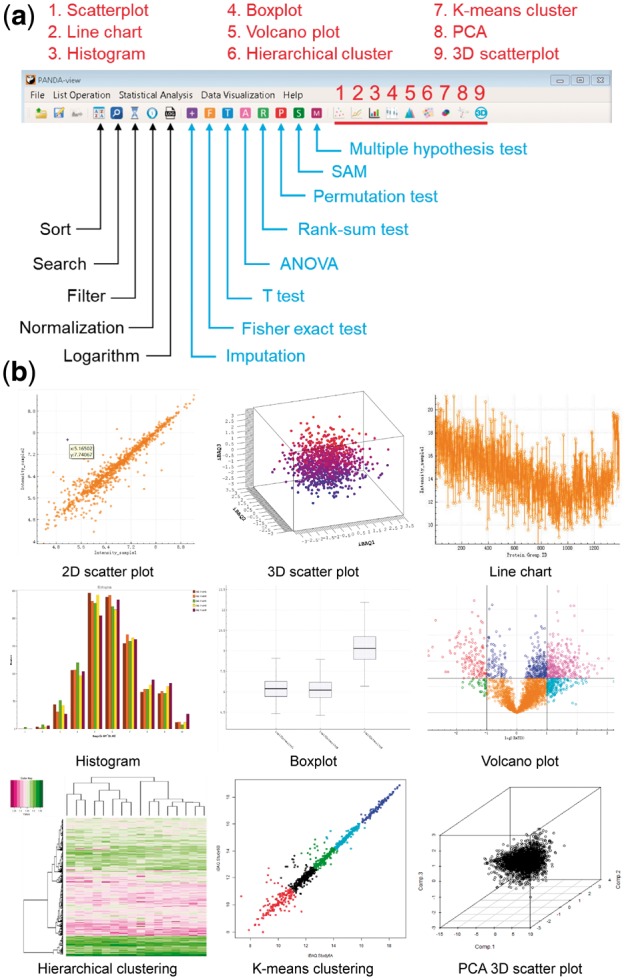
Illustrations of data analysis and visualization functions in PANDA-view. (**a**) Icons of the analysis and visualization functions in the menu. (**b**) Examples of data visualizations

PANDA-view is designed to provide comprehensive methods for statistical analysis and visualization of -omics data, including the quantitative proteomics data (Fig. 1). See Supplementary Note for the detailed descriptions of every function in PANDA-view.

### 2.1 Data upload and pre-process

The input data of PANDA-view can be any CSV or tab-delimited text file obtained from other tools. Once a file is chosen, all its column names will be shown in a wizard graphical user interface (GUI). Users can choose specific columns to load into PANDA-view. Further, when reading extremely large files, multi threads will be automatically started and the uploaded data can be displayed in the GUI in dynamic real time to avoid potential halt or crash. PANDA-view includes five kinds of operations for users to explore and pre-process their data: (i) Sort any column by numerical or character value using an efficient quick sort algorithm (with the median-of-three strategy). (ii) Search any column using user-defined keys. (iii) Filter any column with user-defined parameters. (iv) Logarithm. (v) Normalization, i.e. Z-score normalization, median normalization, maximum normalization, global normalization, interquartile range normalization, quantile normalization and variance stabilization normalization (Fig. 1a). Users can try different normalization methods and choose a best one for their data (Valikangas *et al.*, 2018).

### 2.2 Missing value imputation

It is known that missing value has a detrimental influence on the analysis of -omics data, such as DEP detection (Wang *et al.*, 2017). Thus, missing values are usually imputed before future analysis. Based on R statistical environment (https://www.r-project.org/), two missing value imputation methods are implemented in PANDA-view: multiple imputation and K-nearest neighbors (KNN) imputation.

### 2.3 Statistical analysis

As shown in Figure 1a, there are seven kinds of statistical tests in PANDA-view for DEP detection in different situations. (i) Parametric tests: *t* test (paired *t* test, independent *t* test and Welch’s *t* test) and ANOVA. (ii) Non-parametric tests: rank-sum test, permutation test and Fisher exact test. Specially, Fisher exact test is used to analyze discrete value, such as protein spectral counts. (iii) Significance analysis of microarrays (SAM) (Tusher *et al.*, 2001). Although it was originally proposed for microarray data, SAM remains its popularity for -omics data due to its kinds of variants. (iv) Multiple hypothesis test. PANDA-view includes several prevalent methods to adjust the *P*-values, such as the Bonferroni method (Dunn, 1961), the Benjamini–Hochberg method (Benjamini and Hochberg, 1995) and the Benjamini–Yekutieli method (Benjamini and Yekutieli, 2001).

### 2.4 Unsupervised analysis of -omics data

For -omics data, PANDA-view incorporates three popular unsupervised analysis methods, i.e. hierarchical clustering, K-means clustering and principal component analysis (PCA). For PCA, in addition to the scree plot, biplot and prediction plot in 2D, PANDA-view also provides a 3D scatter plot and a 3D biplot for visualization of the principal components. See Figure 1b and Supplementary Figures S1–S4 for details.

### 2.5 Data visualization

Besides the various kinds of data analysis methods, PANDA-view also contains frequently-used visualization methods, including the 2D/3D scatter plot, the line chart, the histogram and the boxplot (Fig. 1b). All these figures can be clicked and dragged to zoom in or out and can be exported as images (JPG/PNG/BMP) or PDF files in user-defined size and resolution. Moreover, PANDA-view implements an interactive volcano plot for DEP detection. Any data column can be searched using user-defined keys and the retrieved results will be highlighted in the volcano plot (Supplementary Fig. S5).

### 2.6 Multi-level representation of proteomic quantification results

PANDA-view has a special feature, i.e. displaying the quantitative analysis results of PANDA in multiple levels. It can automatically recognize PANDA’s outputs (protein/peptide/peptide ion quantification results). By right-clicking the corresponding index in the quantification result file, PANDA-view can track a protein to its quantified peptides and then to the corresponding peptide ions with the extracted ion chromatography (XIC) views. Thus, a multi-level representation of the proteomic quantification results (protein, peptide, peptide ion and XIC) can be performed in PANDA-view, which is expected to help users make an in-depth analysis of their data (Supplementary Fig. S6). Note, peptide ion indicates that the peptide with certain charge and post-translational modification identified by MS.

## 3 Conclusion

In summary, PANDA-view is an easy-to-use and multifunctional tool for statistical analysis and visualization of -omics data, especially the quantitative proteomics data. It can handle both labeled and label-free quantitative data by offering comprehensive methods for data pre-process, DEP statistical test, as well as clustering analysis and PCA. Besides the commonly-used data visualization methods, PANDA-view implements a multi-level representation for the quantification results of PANDA, which is helpful for end users to explore and manually validate their data in detail.

## Funding

This work was supported by the National Key Research and Development Program of China [2017YFA0505002 and 2017YFC0906602] and the National Natural Science Foundation of China [21605159].

*Conflict of Interest*: none declared.

## Supplementary Material

Supplementary Data
