# Supplementary material for: PANDA-view: an easy-to-use tool for statistical analysis and visualization of quantitative proteomics data
Source: Bioinformatics. 2018 May 22;34(20):3594–6. doi: 10.1093/bioinformatics/bty408 (PMC6184437; doi:10.1093/bioinformatics/bty408)
Supplement: Supplementary Data [file bty408_suppl_data.zip › bty408_Supplementary_Figures.docx]

PANDA-view: An easy-to-use tool for statistical analysis and visualization of quantitative proteomics data

Cheng Chang^1,*^, Kaikun Xu^1^, Chaoping Guo^2^, Jinxia Wang^1,3^, Qi Yan^2^, Jian Zhang^2^, Fuchu He^1^ and Yunping Zhu^1,*^

*^1^State key Laboratory of Proteomics, Beijing Proteome Research Center, Beijing Institute of Lifeomics, National Center for Protein Sciences (Beijing), Beijing 102206, P.R. China.*

*^2^Beijing Key Laboratory of Human Computer Interactions, Institute of Software, Chinese Academy of Sciences, Beijing 100190, P.R. China.*

*^3^Drug Research and Development Center, Shandong Drug and Food Vocational College, Weihai, 264210, P.R. China.*

Correspondence should be addressed to:

Cheng Chang (1987ccpacer@163.com)

Yunping Zhu (zhuyunping@gmail.com)

# Supplementary Figures

## Legends of supplementary figures

Supplementary Figure 1. Parameter setting and an example output of hierarchical clustering. (a) Simple mode. (b) Advanced mode. (c) An example output.

Supplementary Figure 2. Parameter setting (left) and the example output (right) of K-means clustering.

Supplementary Figure 3. Parameter setting (left) and the example outputs (right) of PCA in 2D.

Supplementary Figure 4. Parameter setting (left) and the example outputs (right) of PCA in 3D.

Supplementary Figure 5. Parameter setting and the example output of volcano plot. (a) Parameter setting for volcano plot. (b) Illustration of the interactivity in volcano plot in which data can be searched using user-defined keys and the retrieved results will be highlighted.

Supplementary Figure 6. Multi-level representation of quantitative data in PANDA-view. (a) GUI for uploading data. When loading the results of PANDA, the file types (Protein/Peptide/Peptide ion quantification results) will be automatically recognized. (b) Illustration of a multi-level representation of quantitative data: protein list 🡪 peptide list 🡪 peptide ion list 🡪 XIC view.


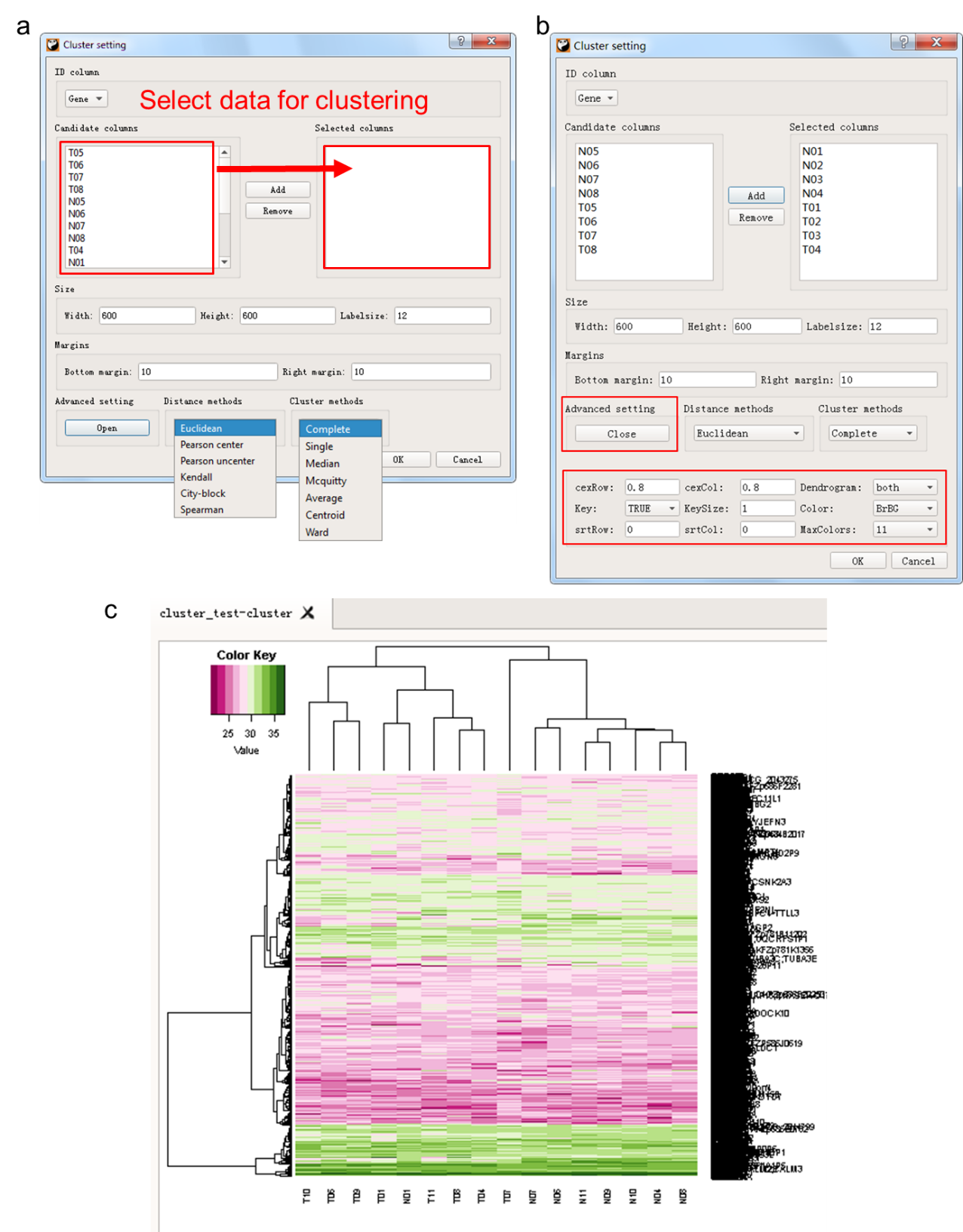


Supplementary Figure 1. Parameter setting and an example output of hierarchical clustering. (a) Simple mode. (b) Advanced mode. (c) An example output.


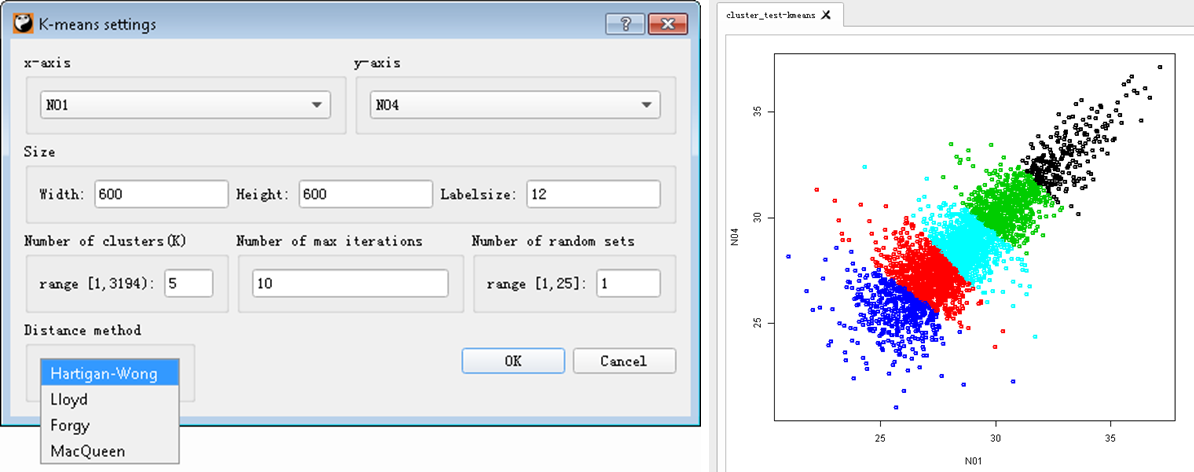


Supplementary Figure 2. Parameter setting (left) and the example output (right) of K-means clustering.


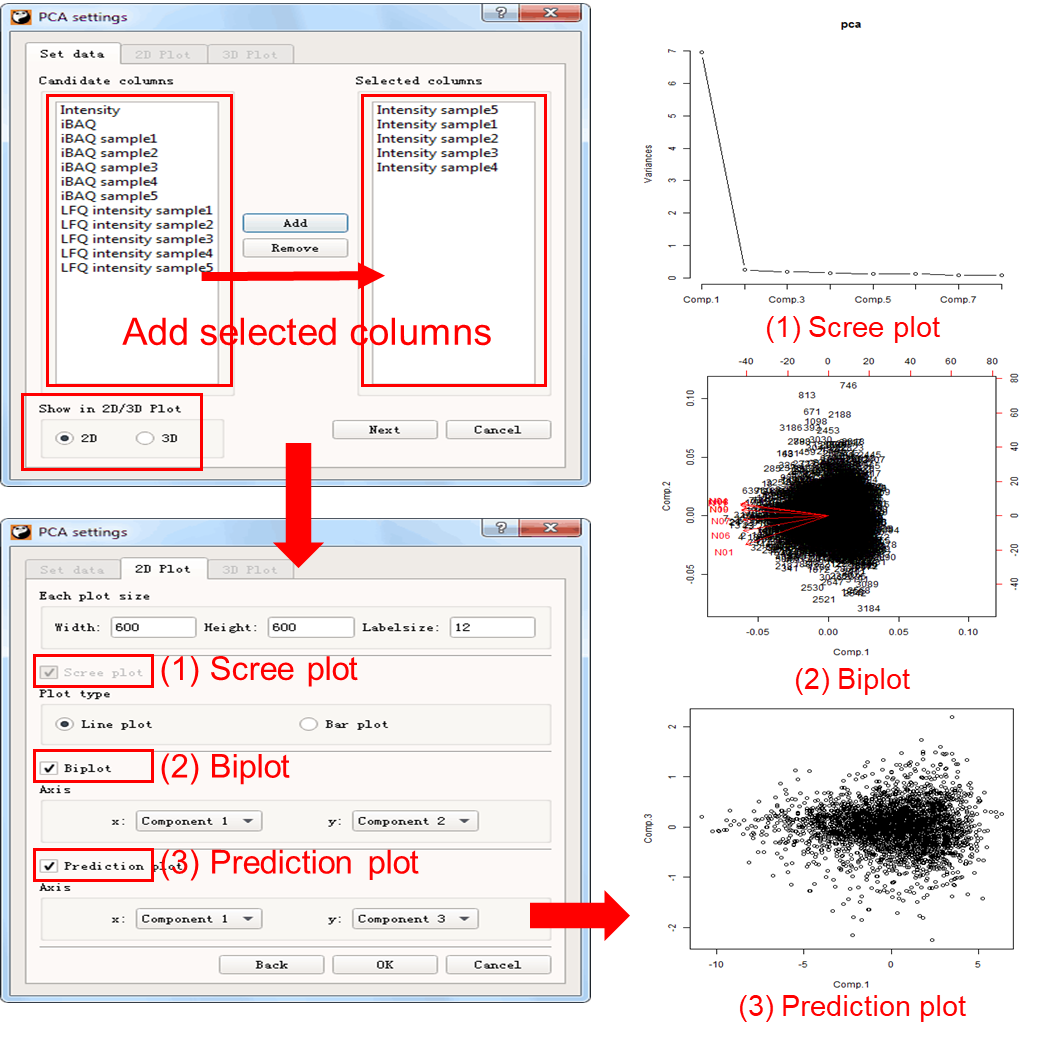


Supplementary Figure 3. Parameter setting (left) and the example outputs (right) of PCA in 2D.


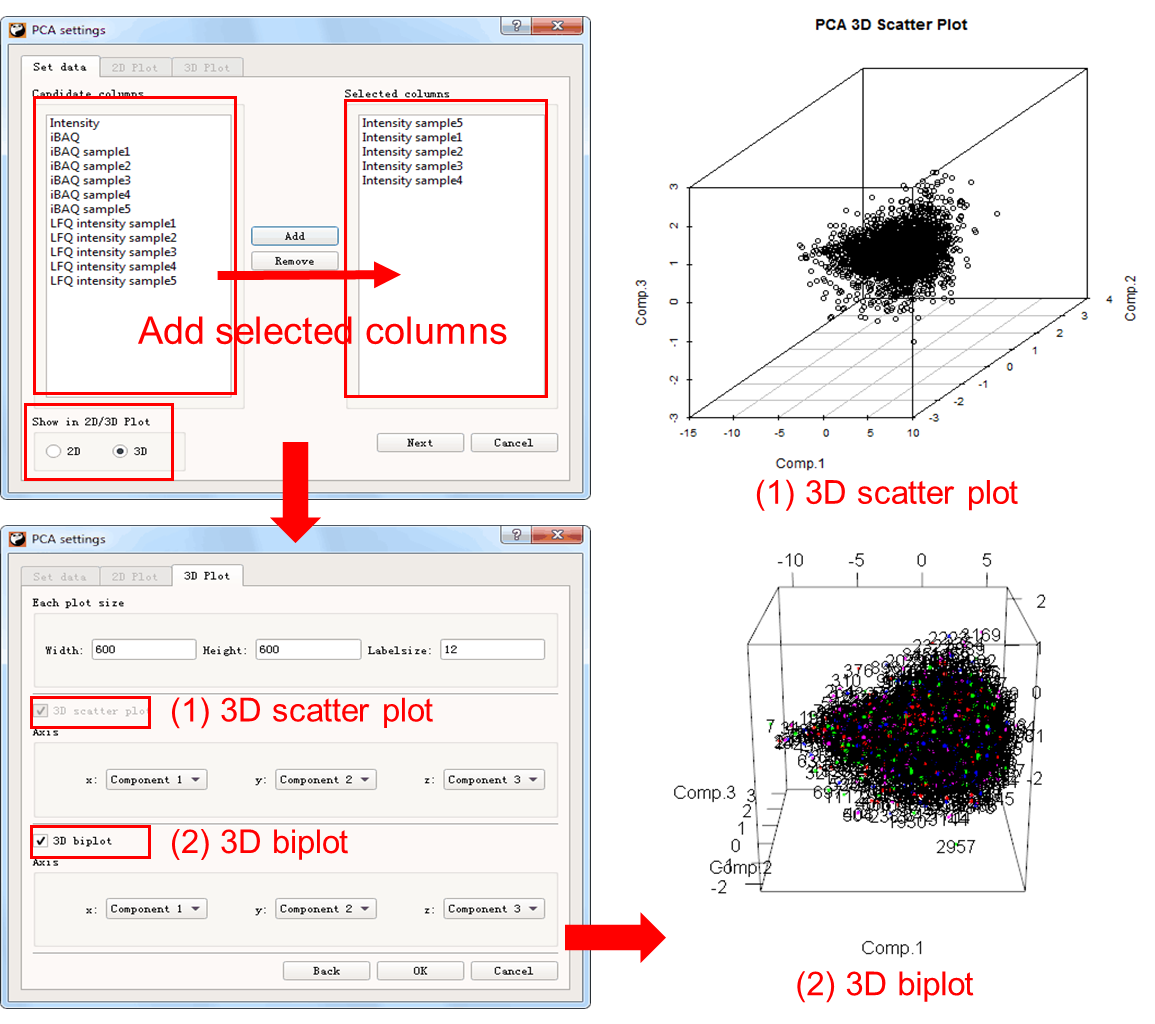


Supplementary Figure 4. Parameter setting (left) and the example outputs (right) of PCA in 3D.


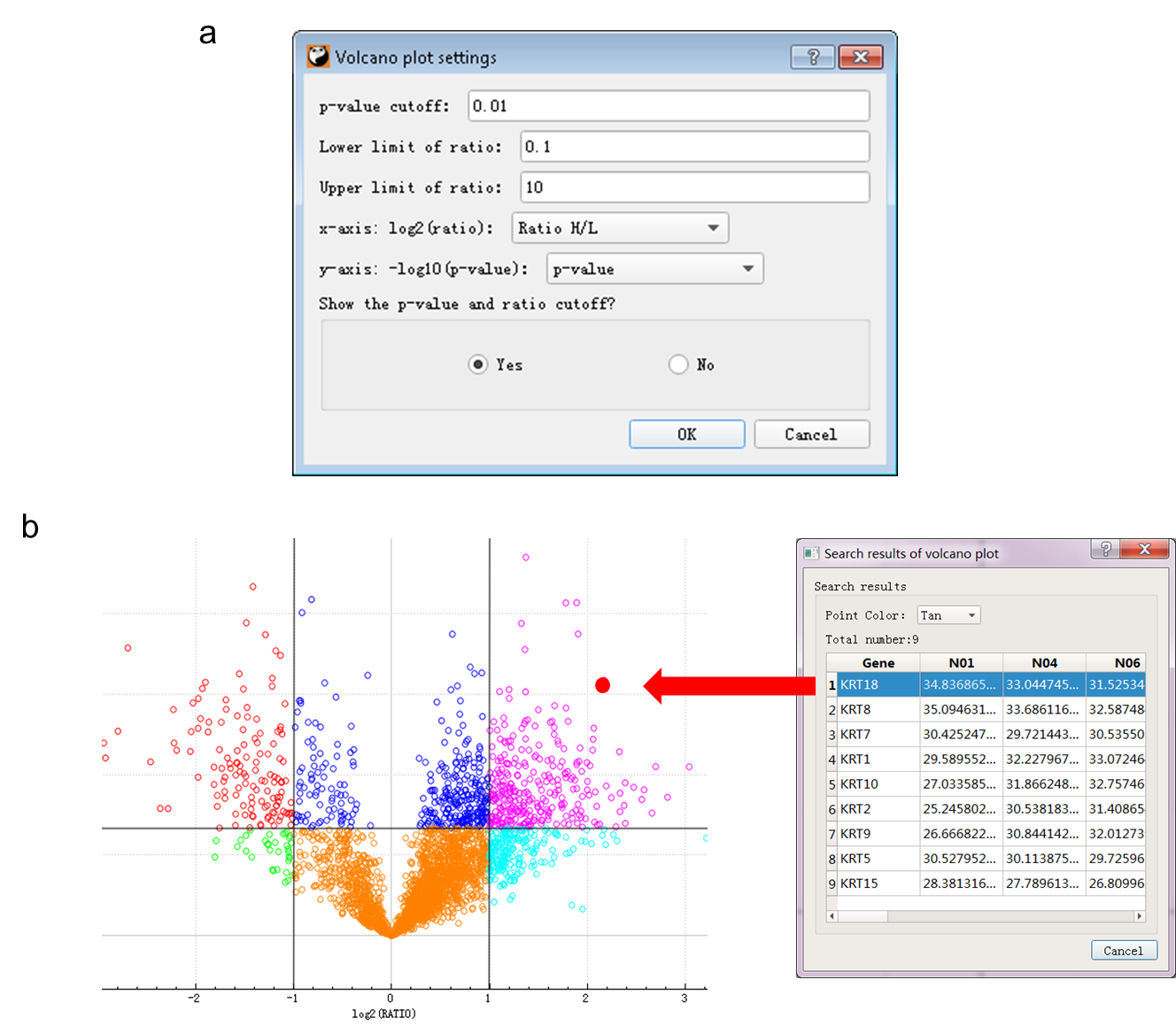


Supplementary Figure 5. Parameter setting and the example output of volcano plot. (a) Parameter setting for volcano plot. (b) Illustration of the interactivity in volcano plot in which data can be searched using user-defined keys and the retrieved results will be highlighted.


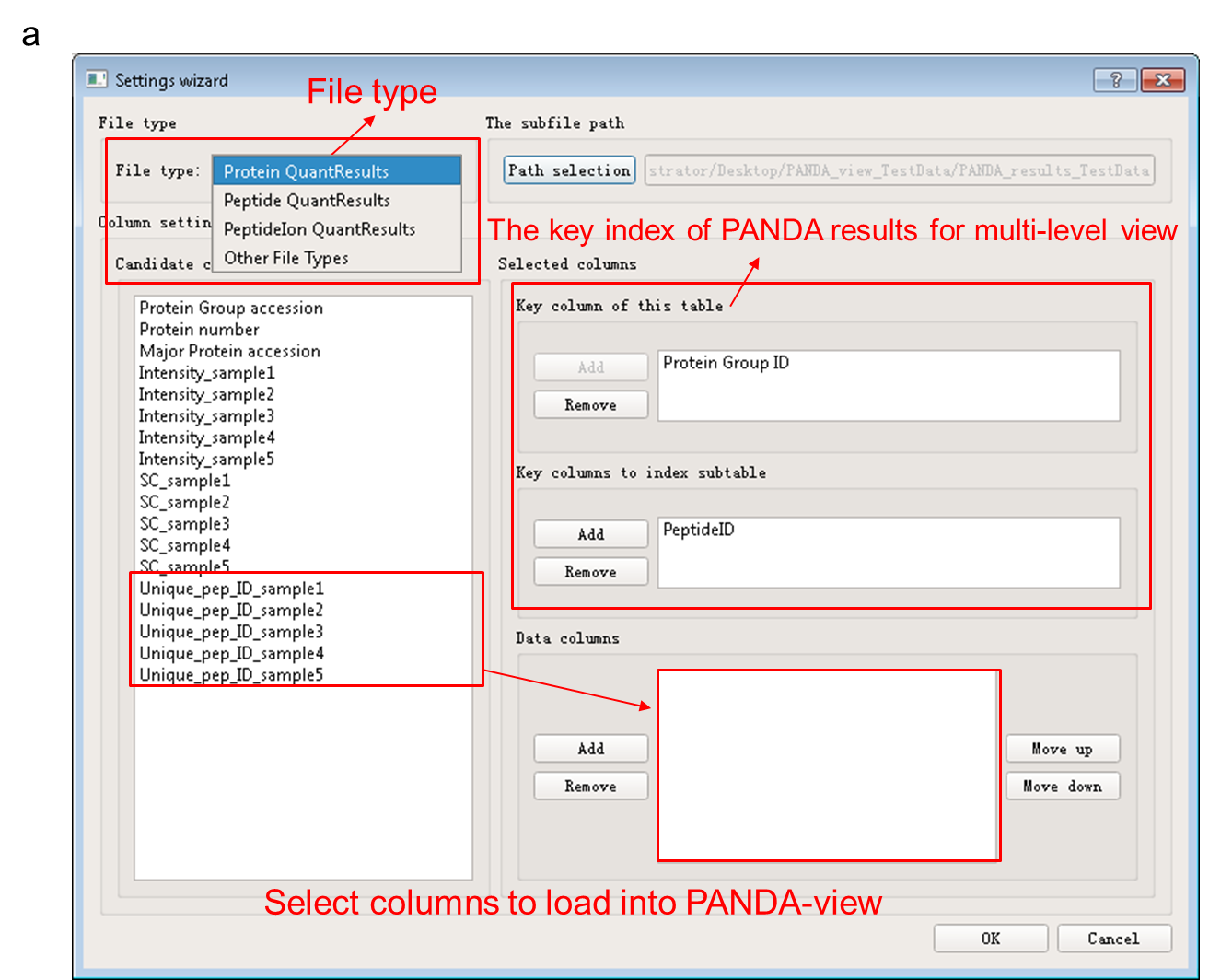


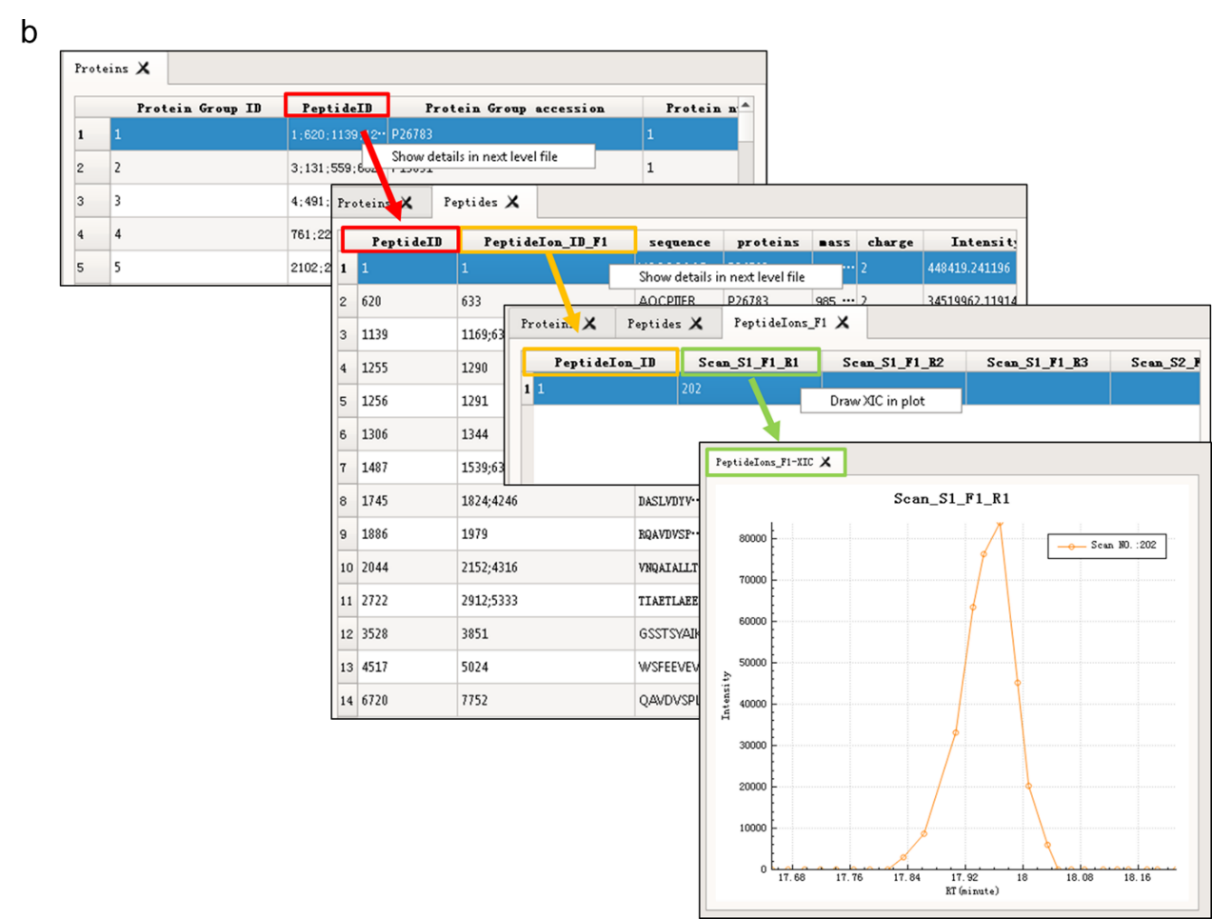


Supplementary Figure 6. Multi-level representation of quantitative data in PANDA-view. (a) GUI for uploading data. When loading the results of PANDA, the file types (Protein/Peptide/Peptide ion quantification results) will be automatically recognized. (b) Illustration of a multi-level representation of quantitative data: protein list 🡪 peptide list 🡪 peptide ion list 🡪 XIC view.
