# Supplementary material for: PANDA-view: an easy-to-use tool for statistical analysis and visualization of quantitative proteomics data
Source: Bioinformatics. 2018 May 22;34(20):3594–6. doi: 10.1093/bioinformatics/bty408 (PMC6184437; doi:10.1093/bioinformatics/bty408)
Supplement: Supplementary Data [file bty408_suppl_data.zip › bty408_Supplementary_Note.docx]

|   An easy-to-use tool for statistical analysis and visualization of quantitative proteomics data | **User Manual for PANDA-view**  Author: Dr. Cheng Chang  Address: State Key Laboratory of Proteomics, Beijing Proteome Research Center, Beijing 102206, China |
| --- | --- |

**Contents**

[Chapter 1. Software Overview 1](#_Toc512705117)

[Chapter 2. Installation 2](#_Toc512705118)

[2.1 Requirement 2](#_Toc512705119)

[2.2 Configuration of R Environment 2](#_Toc512705120)

[2.2.1 Setting system environment variable 2](#_Toc512705121)

[2.2.2 Installing R packages 3](#_Toc512705122)

[2.3 Download 4](#_Toc512705123)

[Chapter 3. Main Functions 6](#_Toc512705124)

[3.1 File 6](#_Toc512705125)

[3.1.1 Open file 6](#_Toc512705126)

[3.1.2 Save table as file 7](#_Toc512705127)

[3.1.3 Export image 7](#_Toc512705128)

[3.1.4 Exit 8](#_Toc512705129)

[3.2 List Operations 8](#_Toc512705130)

[3.2.1 Sort 8](#_Toc512705131)

[3.2.2 Search 9](#_Toc512705132)

[3.2.3 Filter 10](#_Toc512705133)

[3.2.4 Normalization 11](#_Toc512705134)

[3.2.5 Logarithm 12](#_Toc512705135)

[3.3 Statistical Analysis 13](#_Toc512705136)

[3.3.1 Imputation 14](#_Toc512705137)

[3.3.2 Fisher exact test 14](#_Toc512705138)

[3.3.3 T test 16](#_Toc512705139)

[3.3.4 ANOVA 18](#_Toc512705140)

[3.3.5 Rank-sum test 19](#_Toc512705141)

[3.3.6 Permutation test 21](#_Toc512705142)

[3.3.7 SAM 22](#_Toc512705143)

[3.3.8 Multiple hypothesis test 23](#_Toc512705144)

[3.4 Unsupervised Analysis 24](#_Toc512705145)

[3.4.1 Hierarchical clustering 24](#_Toc512705146)

[3.4.2 K-means clustering 25](#_Toc512705147)

[3.4.3 PCA 26](#_Toc512705148)

[3.5 Data Visualization 28](#_Toc512705149)

[3.5.1 2D Scatterplot 28](#_Toc512705150)

[3.5.2 3D Scatterplot 29](#_Toc512705151)

[3.5.3 Line chart 30](#_Toc512705152)

[3.5.4 Histogram 31](#_Toc512705153)

[3.5.5 Boxplot 32](#_Toc512705154)

[3.5.6 Volcano plot 33](#_Toc512705155)

[3.6 Multi-level Representation of Quantitative Data 34](#_Toc512705156)

[3.7 Help 36](#_Toc512705157)

[3.7.1 Language 36](#_Toc512705158)

[3.7.2 R requirement 37](#_Toc512705159)

[3.7.3 About us 39](#_Toc512705160)

[Chapter 4. Support Services 40](#_Toc512705161)

[4.1 Contact 40](#_Toc512705162)

[4.2 Copyright 40](#_Toc512705163)

[Chapter 5. References 41](#_Toc512705164)

# Chapter 1. Software Overview

PANDA-view is developed for statistical analysis and data visualization as an affiliated tool of [PANDA](https://sourceforge.net/projects/panda-tools/). PANDA-view can directly read and perform a multi-level representation of PANDA’s quantification results. In addition, PANDA-view is compatible with other -omics tools by taking their results in tab-delimited text (*.txt) and CSV (*.csv) file formats as input. The core of PANDA-view was written in [Qt C++ language](http://wiki.qt.io/About_Qt) on the platform of Microsoft Visual Studio ultimate 2013 under Windows System. Parts of the functions in PANDA-view are based on [R statistical environment](https://www.r-project.org/).

# Chapter 2. Installation

The chapter explains how to download and install PANDA-view on the user’s computer.

## 2.1 Requirement

1. Hardware requirements
2. Intel Pentium III/800 MHz or higher (or compatible) although one should probably not go below a dual core processor.
3. 2 GB RAM minimum.
4. Software requirements
   1. Supported operating system (OS) versions (32-bit or 64-bit)

Windows 7 SP1

Windows Server 2008 R2 SP1

Windows Server 2008 SP2

Windows Server 2012 R2

Windows 8

Windows 10

- 1. [.NET Framework 4.5](https://www.microsoft.com/en-us/download/details.aspx?id=30653) or higher from Microsoft.
  2. Microsoft Visual C++ Redistributable for Visual Studio 2013: download from [here](https://www.microsoft.com/en-us/download/details.aspx?id=40784).
  3. R v3.1.2 or higher (for Windows) from [R project](https://www.r-project.org/).

## 2.2 Configuration of R Environment

### 2.2.1 Setting system environment variable

After installing R, users should add the path of RScript.exe into the system environment variable before using PANDA-view. Because PANDA-view implements some R-based methods by calling Rscript.exe to execute the R codes. When there are several versions of R installed in a user’s computer, PANDA-view will call the Rscript.exe whose path is added into the system environment variable. The method for setting system environment variable can be found at <http://www.computerhope.com/issues/ch000549.htm>.

By default, RScript.exe is in the path such as “c:\Program Files\R\R-3.3.3\bin\”. Then, this path should be added into the system environment variable. In addition, the path “c:\Program Files\R\R-3.3.3\bin\x64\” should also be added for the 64-bit OS. See Fig. 1 for details.


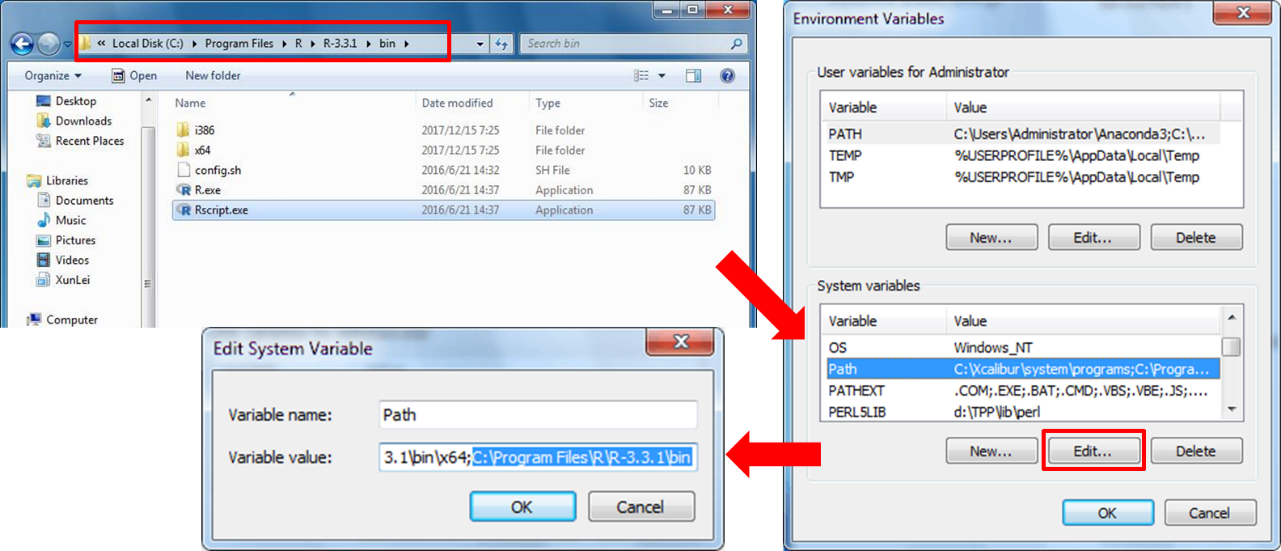


Figure 1. Illustration of adding the RScript.exe path into system environment variable.

### 2.2.2 Installing R packages

The required R packages and their installation commands are listed below:

install.packages("rgl")
install.packages("scatterplot3d")
install.packages("RColorBrewer")
install.packages("gplots")
install.packages("survival")
install.packages("coin")
install.packages("Rcpp")
install.packages("lattice")
install.packages("mice")
source("http://bioconductor.org/biocLite.R")
biocLite("Biobase")
biocLite("limma")
biocLite("impute")
biocLite("R.methodsS3")
biocLite("matrixStats")
biocLite("samr")

Users should install these R packages before starting PANDA-view. Users are recommended to install these packages one by one in case there are dependent packages required to install and click the “R requirement” button in the Help menu to check if the R environment and required R packages are correctly configured and installed (see 3.7.2). See the configuration video at <https://sourceforge.net/projects/panda-view/> for details.

In PANDA-view, the R code will be automatically generated and saved in a fixed file folder (i.e., “c:\temp\RScript\”) after a R-based analysis method is performed. The input and output of a R-based method will be saved in another fixed file folder “c:\temp\RData\”. Thus, users can easily find how their data were analyzed in PANDA-view. Users can directly run these R codes in R environment to facilitate reproducibility.

## 2.3 Download

PANDA-view can be freely downloaded from <https://sourceforge.net/projects/panda-view/>. Un-compress the zip package (or 7z) into a specified file folder. Double-click “PANDA-view.exe” and the graphical user interface (GUI) of PANDA-view will be shown in Fig. 2.


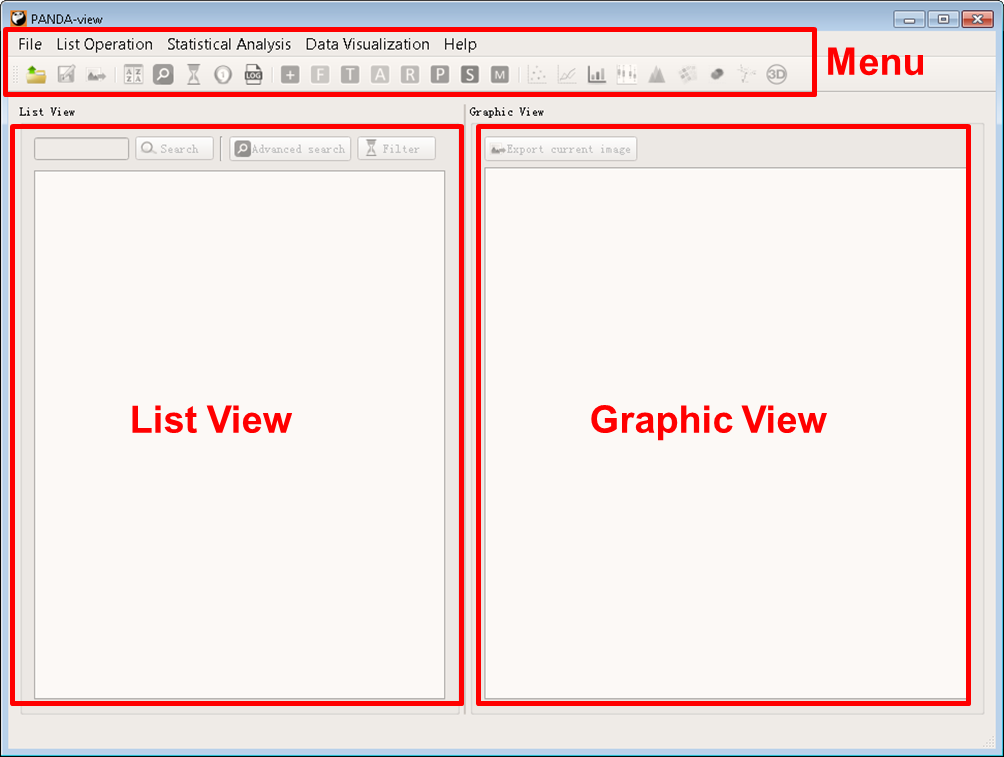


Figure 2. The GUI of PANDA-view.

# Chapter 3. Main Functions

## 3.1 File

As shown in Fig. 3, there are three functions in the drop-down menu of *File*, i.e., *Open file, Save current table as,* and *Export current image as*.


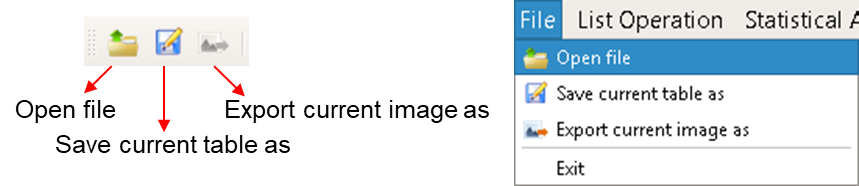


Figure 3. Shortcuts and drop-down menu of *File*.

### 3.1.1 Open file

Users can upload data by clicking the *Open file* item in *File* or click the *Open file* button in *Menu*. Both txt and csv files are supported (Fig. 4).


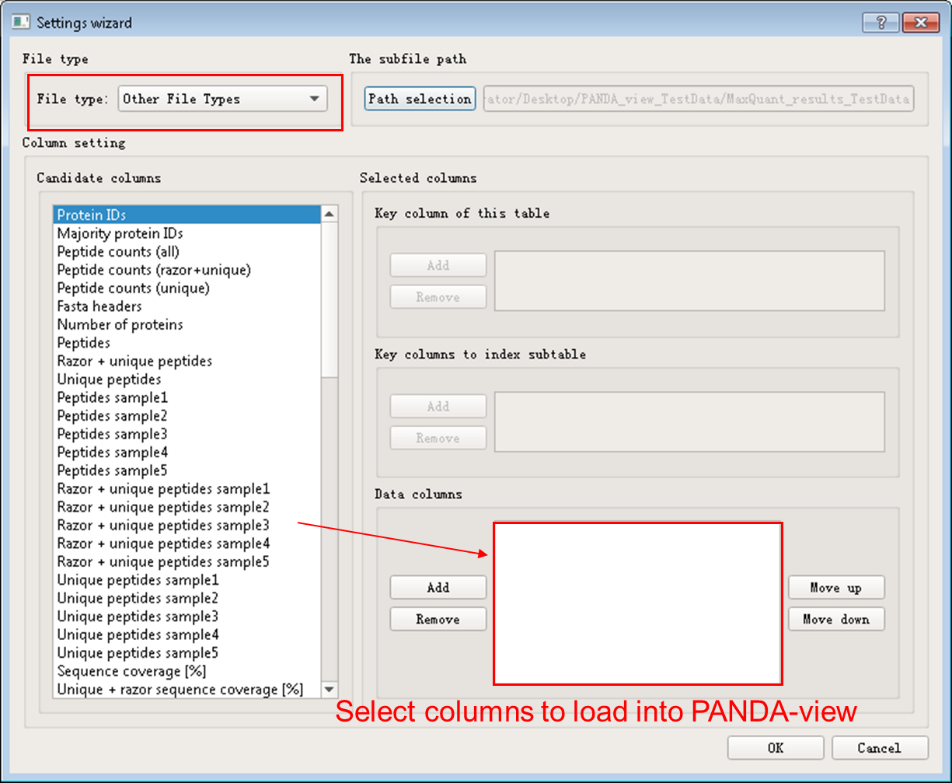


Figure 4. File upload. When loading the results of other tools except PANDA, PANDA-view will consider the data type as “Other File Types”.

Users can choose the data columns from the candidate columns for subsequent analysis. Click *OK* to display the selected data. Additionally, PANDA-view can read the results of PANDA directly, see Chapter 3.6 for details.

### 3.1.2 Save table as file

Users can save the current table in the GUI in the format of *txt* or *csv* by clicking the *save current table as* item in *File* or click the *Save* button in the GUI (Fig. 5).


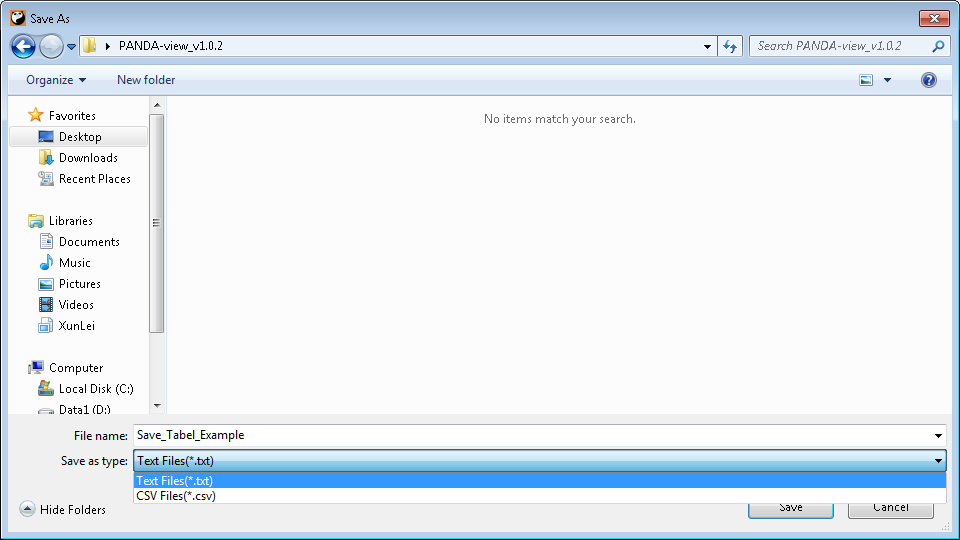


Figure 5. Operations of saving table in PANDA-view.

### 3.1.3 Export image

Users can save the current image in Graphic View of GUI by clicking *Export current image as* item in *File* or clicking the same button in *Menu*. In *Export settings* dialog (Fig. 6), users can set the image size and the resolution with *default resolution, standard resolution, high resolution* and *ultra resolution*. Images can be saved in *PNG, JPG,* *BMP* or *PDF* format.


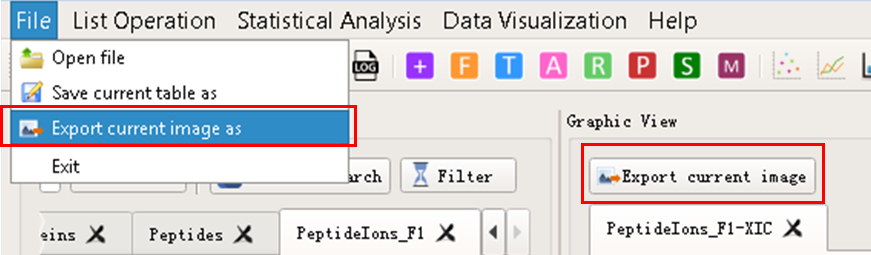


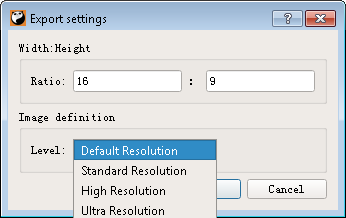


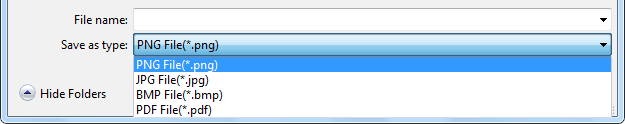


Figure 6. Operations of exporting images in PANDA-view.

### 3.1.4 Exit

To close PANDA-view, users can click the *Exit* item in *File* or click the close symbol at the top right corner of the GUI and a dialog will be shown (Fig. 7). Click *OK* to close PANDA-view or click *Cancel* to stay in PANDA-view.


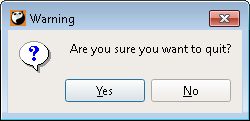


Figure 7. The dialog of exiting PANDA-view.

## 3.2 List Operations

As shown in Fig. 8, there are five functions in the drop-down menu of *List Operation*, i.e., *Sort, Search, Filter, Normalization* and *Logarithm*.


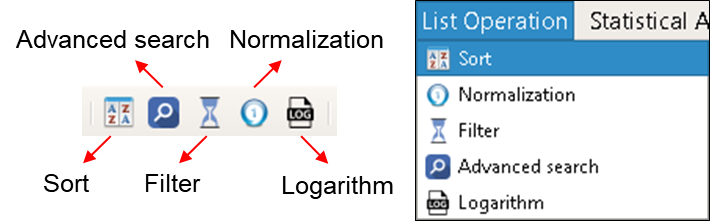


Figure 8. Shortcuts and drop-down menu of *List Operation*.

### 3.2.1 Sort

Clicking the *Sort* item in *List Operation*, users can sort the table in an ascending or descending order. By default, a new data table will be generated after sorting (Fig. 9).


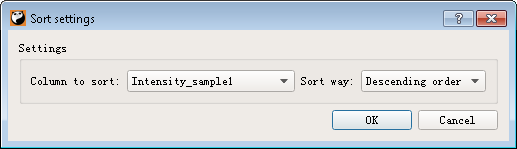


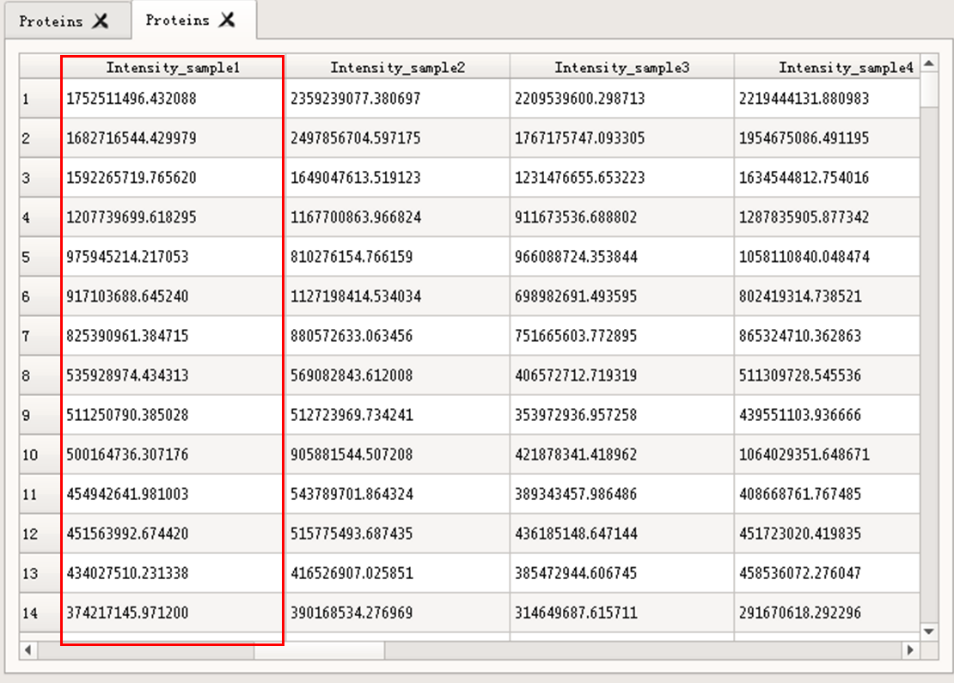


Figure 9. Parameter setting and the example results of sort operation.

### 3.2.2 Search

Users can search key words through the built-in *Search* and *Advanced search* functions. PANDA-view will search the key words in the whole table when the *Search* function is enabled, and this function is not case sensitive by default. Users can click the *Advanced search* item in *List Operation* or click the shortcut in *List view* in *Menu* to make a case-sensitive search or search specific columns (Fig. 10 and Fig. 11). A new table will be generated after searching.


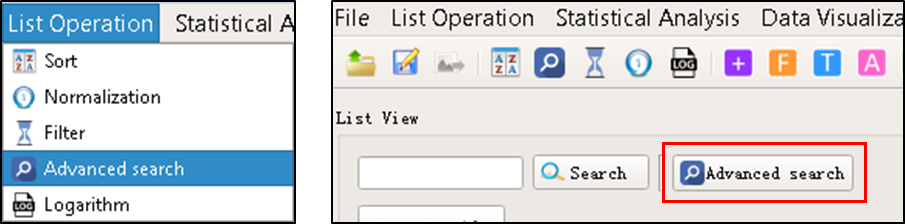


Figure 10. The shortcuts of advanced search in the menu and GUI.


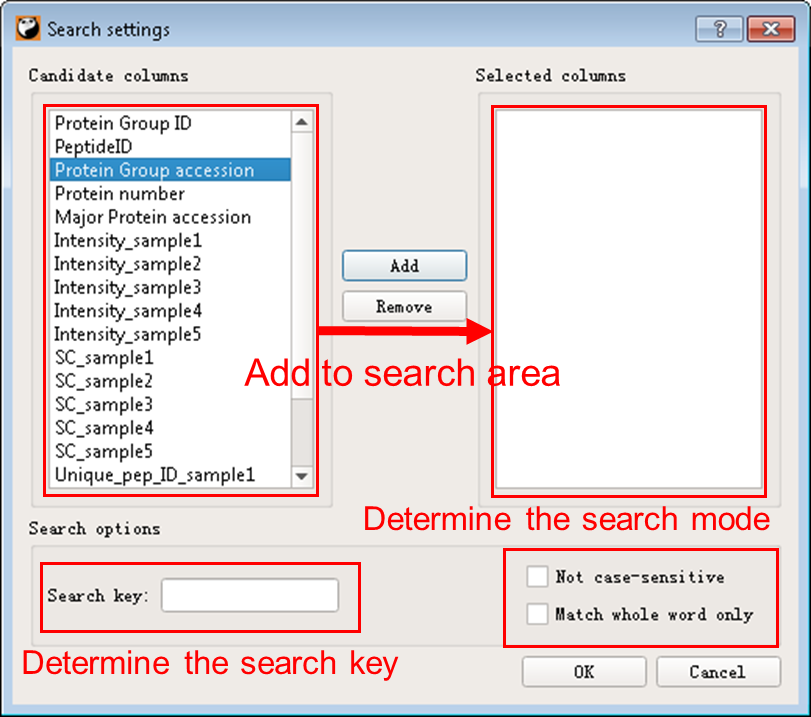


Figure 11. Parameter setting for advanced search.

### 3.2.3 Filter

For numeric columns, users can use *Filter* function to select the values between the user-defined limits by clicking *Filter* item in *List Operation* or click the shortcuts in *Menu*. The built-in function supports multi-column filtering by clicking *add* or *delete* in Filter settings (Fig. 12). A new table will be generated after the operation.


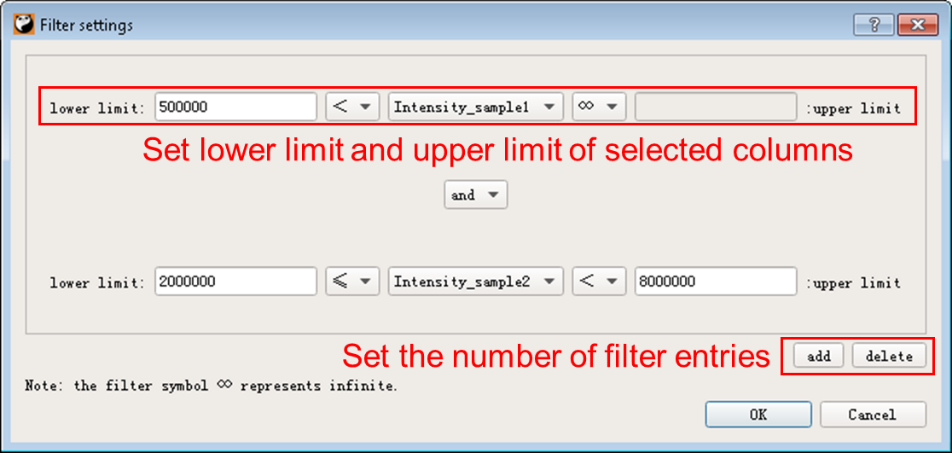


Figure 12. Parameter setting for filtering.

### 3.2.4 Normalization

Normalization is used to exclude sample deviations and adjust values measured on different scales to a common scale. PANDA-view supports the seven normalization methods (Z-score normalization, Median normalization, Maximum normalization, Global normalization, IQR normalization, Quantile normalization and VSN) for numeric columns. The *Normalization* item is in *List Operation* drop-out menu (Fig. 13). Detailed descriptions for every method are as follows:

(1) Z-score normalization. The absolute value of z, also called standard score, is calculated as follows. The mean of $x_{norm}$ is zero and the standard deviation is one.

$$x_{norm}=\frac{x-mean\left( x \right)}{std\left( x \right)}, mean\left( x \right)= \frac{1}{n}\sum_{i=1}^{n} x_{i}, std(x)=\sqrt{\frac{\sum_{i=1}^{n} {(x_{i}-mean(x))}^{2}}{n-1}}$$

(2) Median normalization. The median of $x_{norm}$ is one.

$$x_{norm}=\frac{x}{median(x)}, median\left( x \right)= \frac{x_{\left\lfloor\frac{n}{2} \right\rfloor}+x_{\left\lfloor\frac{n}{2}+\frac{1}{2} \right\rfloor}}{2}$$

(3) Maximum normalization. The maximum of $x_{norm}$ is one.

$$x_{norm}=\frac{x}{max(x)}$$

(4) Global normalization. This method is known as feature scaling in some research field. All values of $x$will be brought into the range [0,1].

$$x_{norm}=\frac{x-min(x)}{max(x)-min(x)}$$

(5) IQR normalization. The interquartile range ($IQR$), also known as middle 50%, is a measure of statistical dispersion, being equal to the first quartile ($Q_{1}$) subtracted from the third quartile ($Q_{3}$). The IQR of $x$ will become one after this normalization.

$$x_{norm}=\frac{x}{IQR}=\frac{x}{Q_{3}-Q_{1}}$$

(6) Quantile normalization. This method is a technique for making two distributions identical in statistical properties, which is frequently used in microarray and -omics data analysis ([Valikangas, et al., 2018](#_ENREF_6)). In PANDA-view, quantile normalization is performed using the *normalizeBetweenArrays* function from the R/Bioconductor package *limma* ([Ritchie, et al., 2015](#_ENREF_5)).

(7) Variance stabilization normalization (VSN). This method is popular for microarray data normalization by making sample variance independent of their mean intensities ([Huber, et al., 2002](#_ENREF_4)). In PANDA-view, VSN is performed using the *normalizeVSN* function from the R/Bioconductor package *limma* ([Ritchie, et al., 2015](#_ENREF_5)).


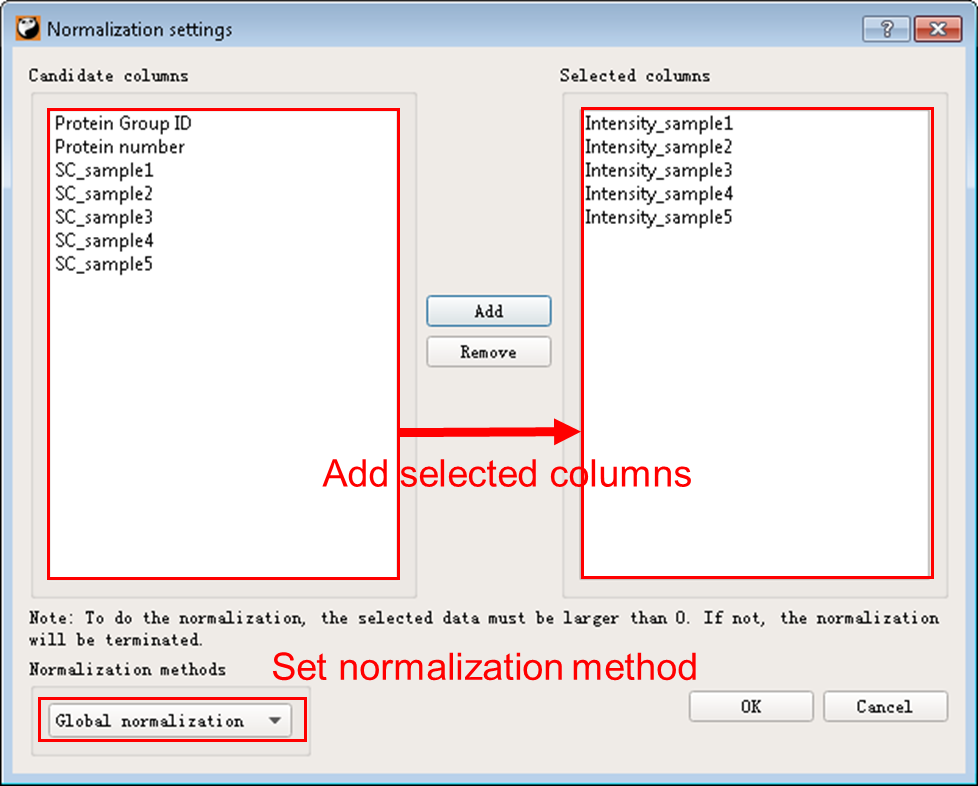


Figure 13. Parameter setting for normalization.

### 3.2.5 Logarithm

Users can calculate the logarithms for numeric columns in which all values are greater than zero by clicking *Logarithms* item in *List Operation* or the corresponding shortcut in *Menu*. Base of logarithms can be set to any positive integer (Fig. 14).


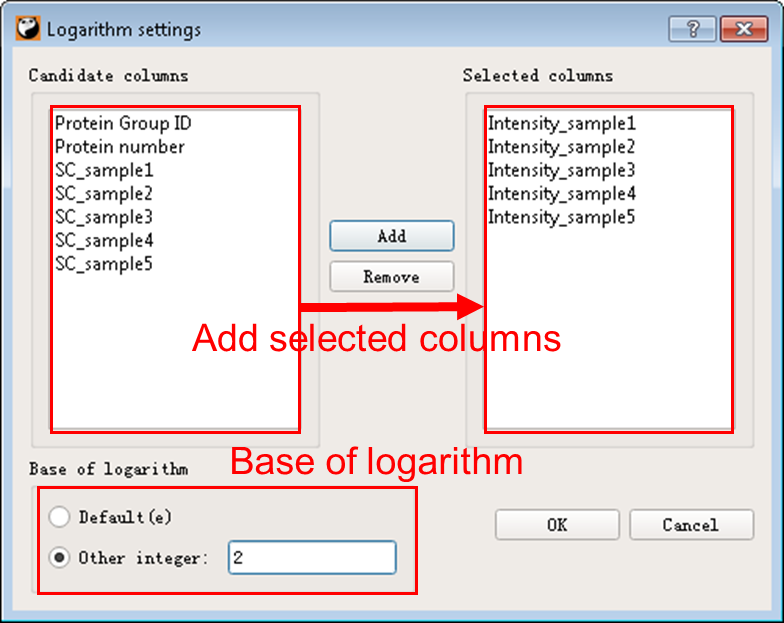


Figure 14. Parameter setting for logarithm.

## 3.3 Statistical Analysis

As shown in Fig. 15, there are two missing value imputation methods and seven kinds of statistical tests implemented in PANDA-view. These methods are all based on R.


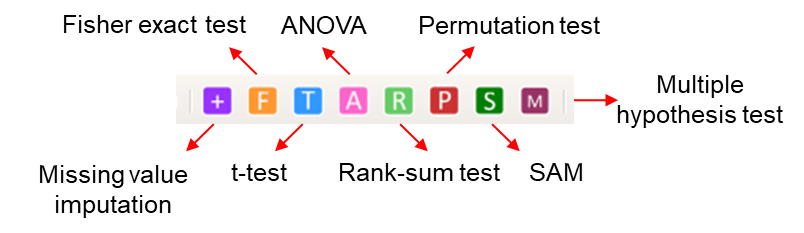


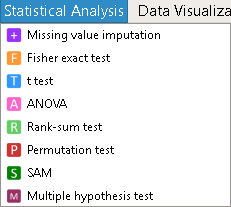


Figure 15. Shortcuts and drop-down menu of *Statistical Analysis*.

### 3.3.1 Imputation

To deal with zero value or NaN (not a number) value, PANDA-view implements two imputation methods: multiple imputation and K-nearest neighbors imputation (KNN imputation) (Fig. 16). For multiple imputation, a logarithmic transformation has been done to transfer zero value to NaN value before interpolation. Due to algorithm requirements, at least two columns of values need to be selected. Especially, PANDA-view will automatically start multiple imputation rather than KNN-imputation if missing percentage of a row is more than 50%. Multiple imputation is based on the *complete* function from R package *mice*. KNN imputation is based on the *impute.knn* function from R/Bioconductor package *impute*.


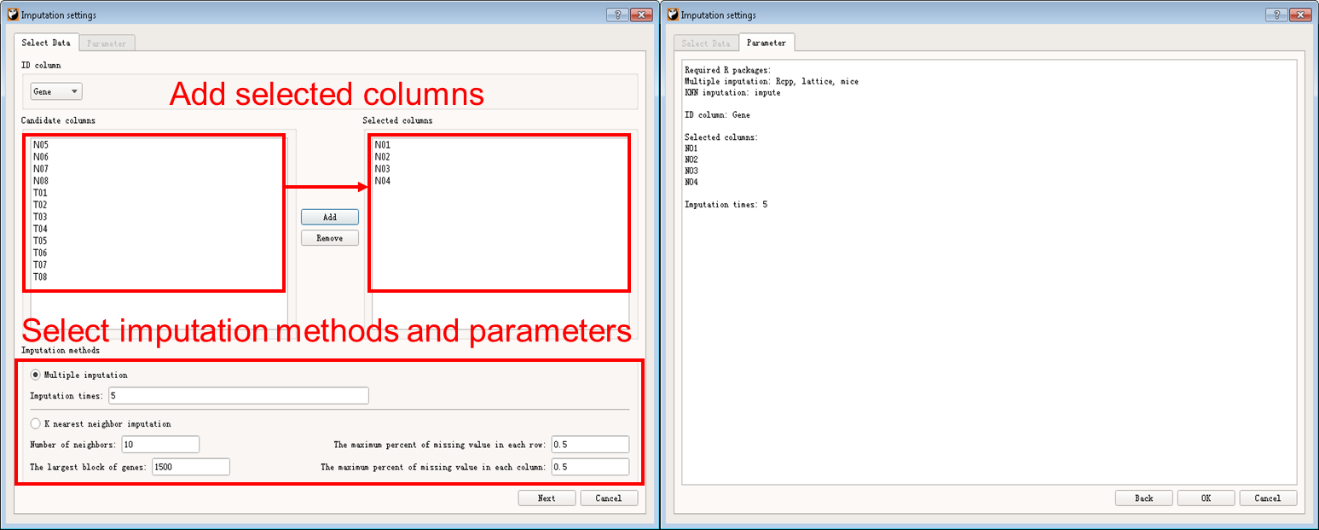


Figure 16. Parameter setting (left) and check (right) for multiple imputation.

### 3.3.2 Fisher exact test

PANDA-view provides *Fisher exact test* for the discrete values of each sample (such as spectral counts data) to test the independence between columns. No missing values (zero, NaN or space) is allowed. The algorithm will determine a fourfold table at first and then calculate the p-value (Table 1). Users can set *Confidence level* (a value between 0 and 1) and *Alternative hypothesis* (two sided, greater or less) (Fig. 17 and Fig. 18). This method is based on the R function *fisher.test*.

Table 1. Fourfold table of one row in Fisher exact test

| Quantitation values | Selected column[i] | Selected column[j] |
| --- | --- | --- |
| Target protein | x | y |
| Other proteins | Sum[i] – x | Sum[j] – y |
| Total | Sum[i] | Sum[j] |


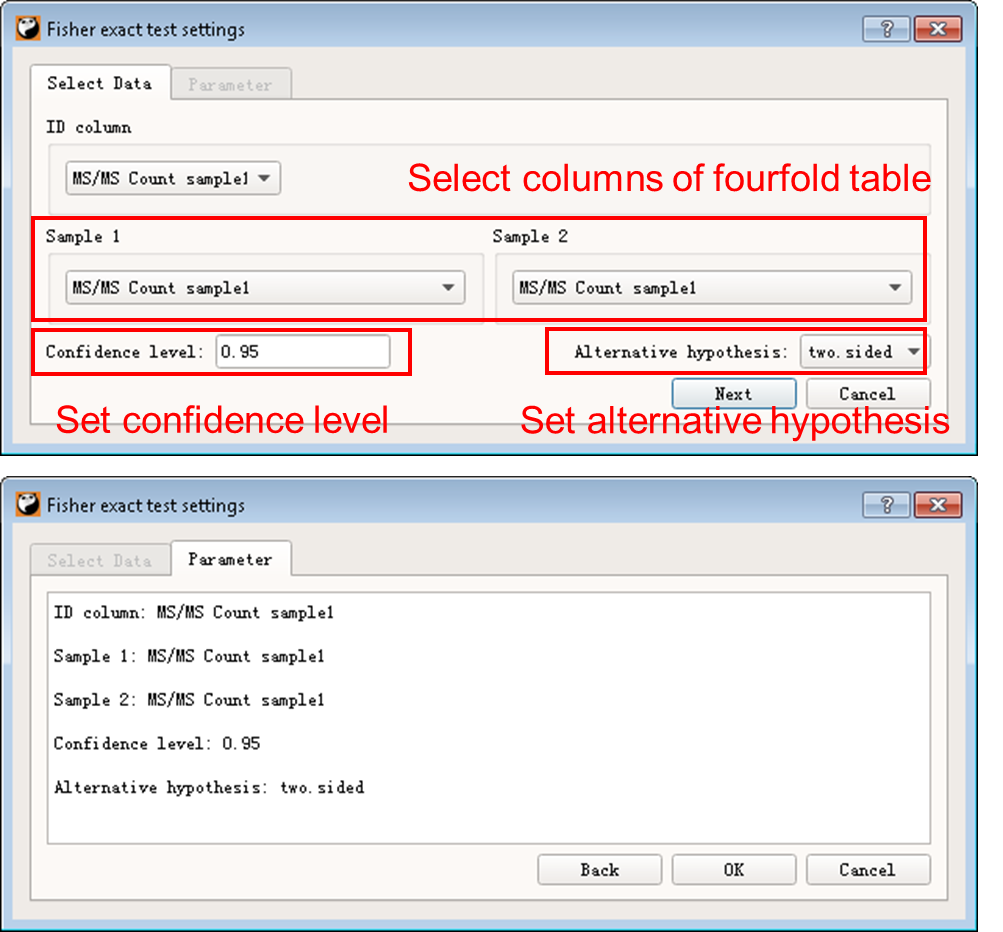


Figure 17. Parameter setting (top) and check (bottom) about Fisher exact test.


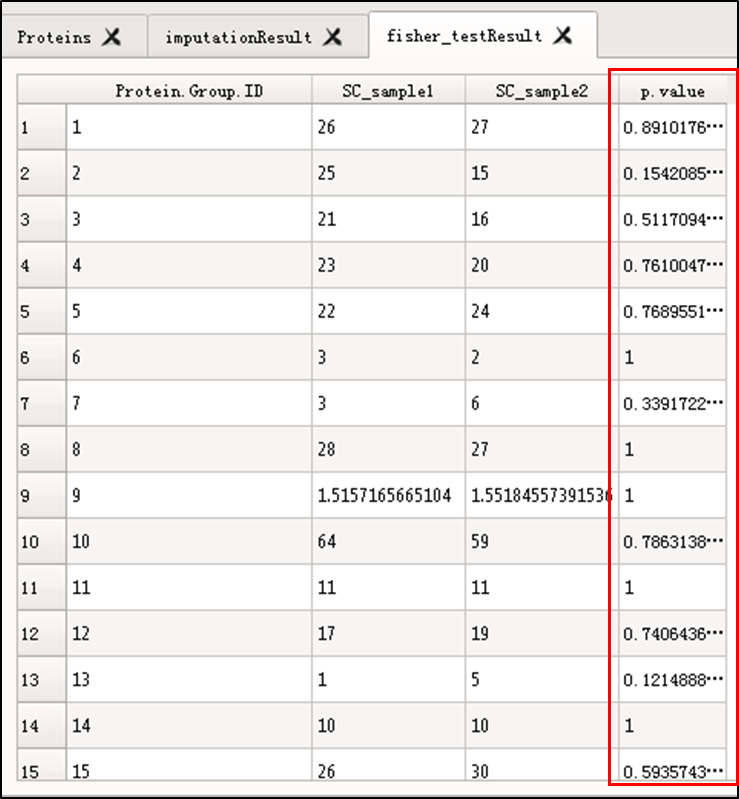


Figure 18. An output example of Fisher exact test.

### 3.3.3 T test

PANDA-view provides *t test* function to judge whether there is a statistical difference of each protein/gene in two samples.

Data need to have an approximately normal distribution to apply t test. Thus, users are recommended to take logarithm of data or perform multiple imputation, in which a logarithmic transformation will be done firstly.

T-test function is enabled when users click the *t test* item in *Statistical Analysis* or the same symbol in *Menu*. In the pop-up window *t test settings*, users can set the *Group* from the candidate columns, *the type of t test* (paired t test, independent t test and Welch’s t test), *Confidence level*, *the mu from null hypothesis* and the *Alternative hypothesis* (two sided, greater or less) to satisfy their own analytical purposes and experimental conditions (Fig. 19 and Fig. 20). This method is based on the R function *t.test*.

It should be noted that paired t test can only be used if two groups come from a paired design in which the sample number in both groups needs to be equal. Besides, for independent t test, data need to meet variance homogeneity. It is recommended to use Welch’s t test if the normality and variance homogeneity of data are unknown (Table 2).

Table 2. An example of t-test for two groups of protein quantitation data

|  | Group1 | | | Group2 | |
| --- | --- | --- | --- | --- | --- |
| Sample | Sample1 | Sample2 | Sample3 | Sample1 | Sample2 |
| Protein[i] | x | y | z | a | b |


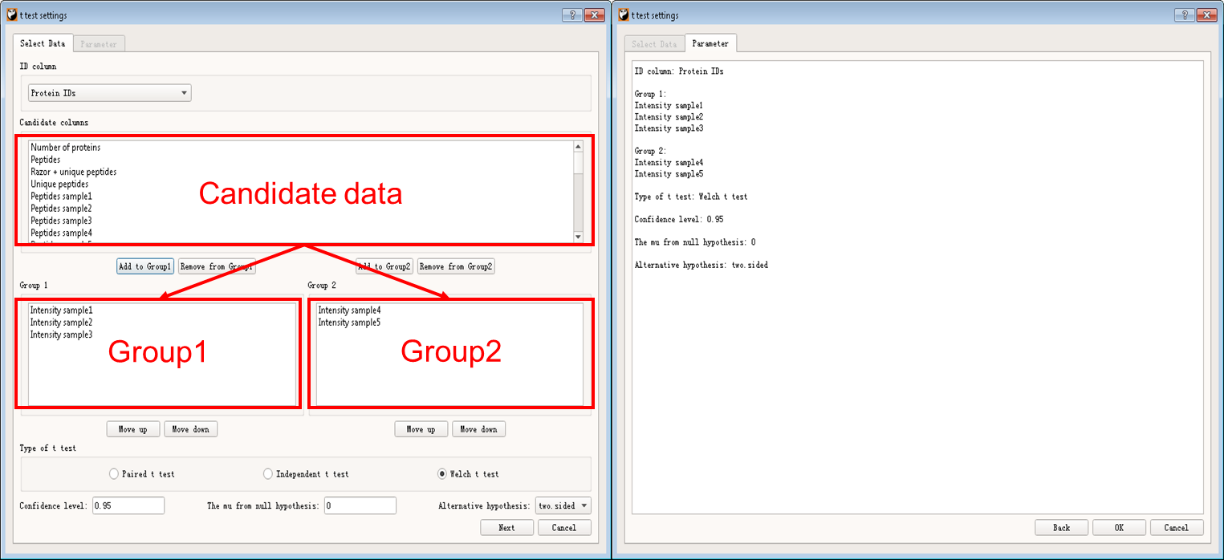


Figure 19. Parameter setting (left) and check (right) for t test.


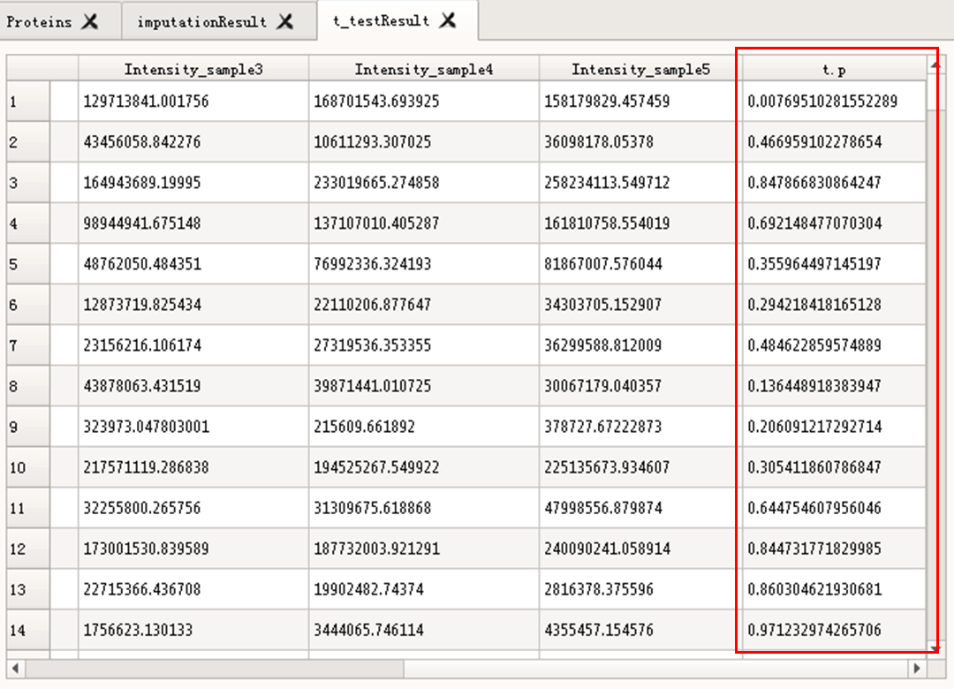


Figure 20. An output example of t test.

### 3.3.4 ANOVA

PANDA-view provides *ANOVA* function to analyze the difference among group means of different experimental conditions in single factor experiment design. Users can activate the function by clicking the *ANOVA* item in *Statistical Analysis* or the same symbol in *Menu*.

The number of samples under each condition must be larger than two but not compulsorily equal. At least three groups are needed to perform ANOVA. Specially, like t-test, users are recommended to take logarithm or perform multiple imputation to make data meet normality. Especially, for the experiments with many samples, users can multi-select data using the “Ctrl” key and click the “Set group” button to set the group IDs of multiple data columns at the same time (Fig. 21). After calculation, PANDA-view will output the p-value for each row (i.e. protein/gene) (Fig. 22). This method is based on the R function *aov*.


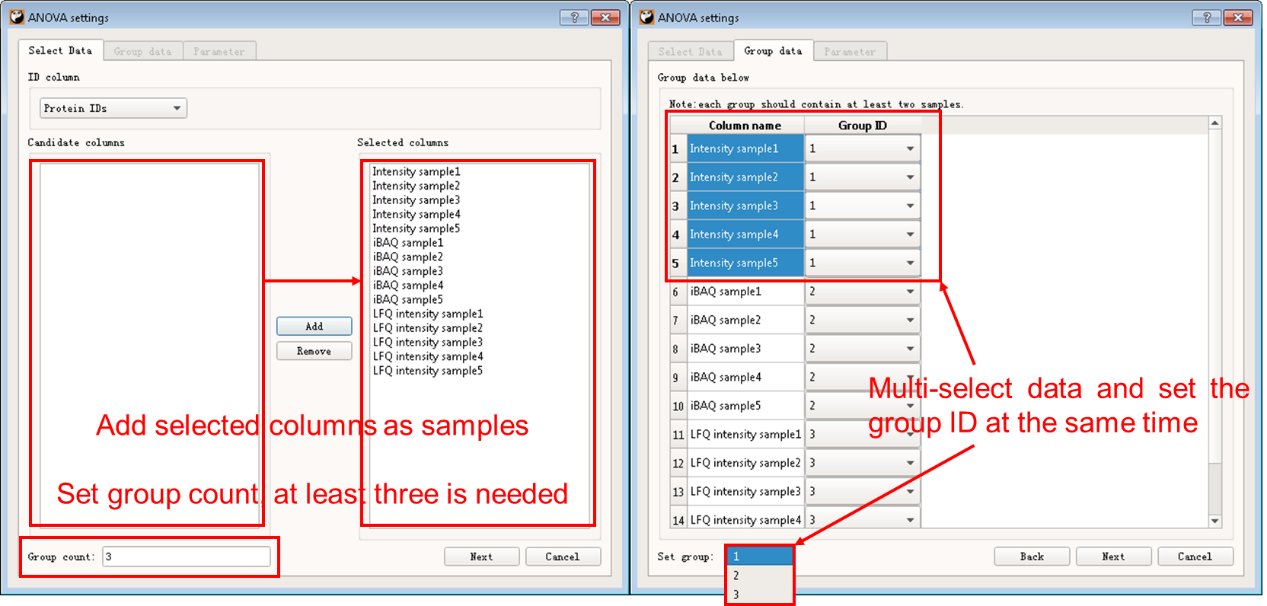


Figure 21. Parameter setting for ANOVA. For the experiments with many samples, users can multi-select data using the “Ctrl” key and click the “Set group” button to set the group IDs of multiple data columns at the same time.


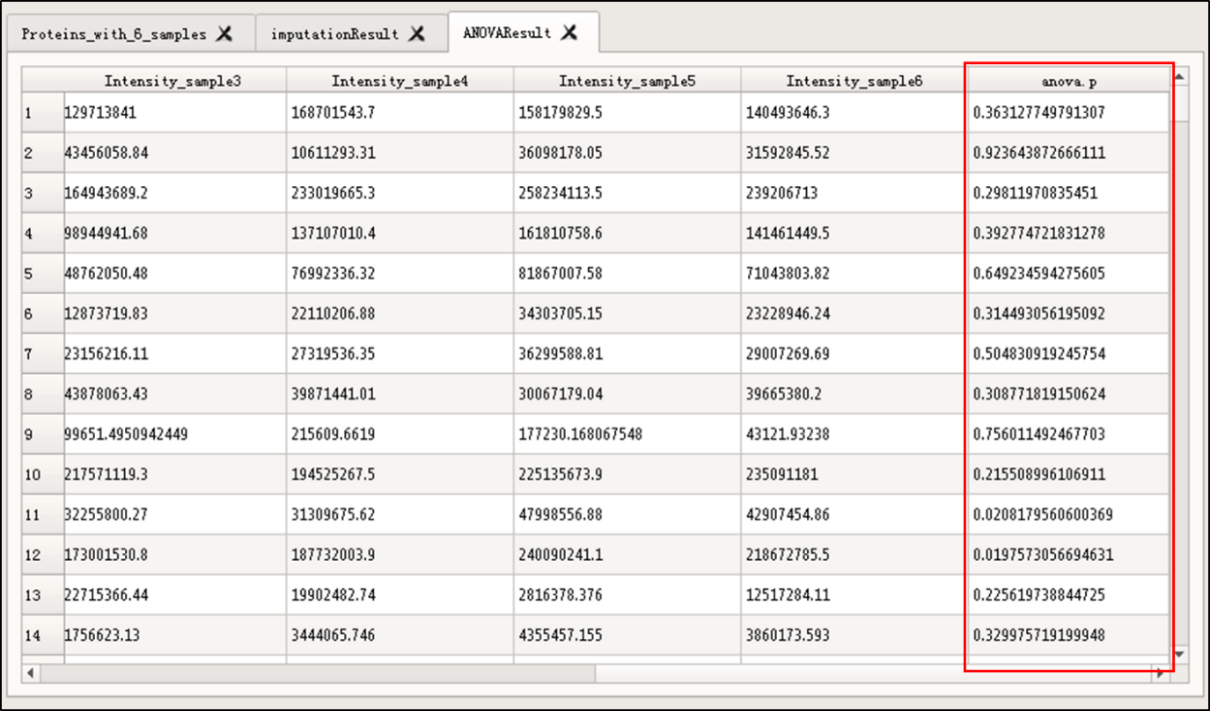


Figure 22. An output example of ANOVA.

### 3.3.5 Rank-sum test

PANDA-view provides *Rank-sum test* function to judge whether samples are originated from the same distribution. There is no requirement for data distribution status in this function. Users can activate the function by clicking the *Rank sum test* item in *Statistical Analysis* or the same symbol in *Menu*.

As shown in Fig. 23 and Fig. 24, users need to set the parameters in *Rank-sum test setting* dialog based on their own experiment conditions. Users are suggested to use *Wilcoxon rank sum test* when they only have two experimental conditions. In contrast, Kruskal-Wallis test by ranks, i.e. Kruskal–Wallis H test or Kruskal–Wallis rank sum test, is recommended to enable when they have more than two experimental conditions. Note that the number of samples in each group needs to be equal when the *Paired* option is set to yes. Wilcoxon rank sum test and Kruskal–Wallis rank sum test are based on the R function *wilcox.test* and *kruskal.test*, respectively.


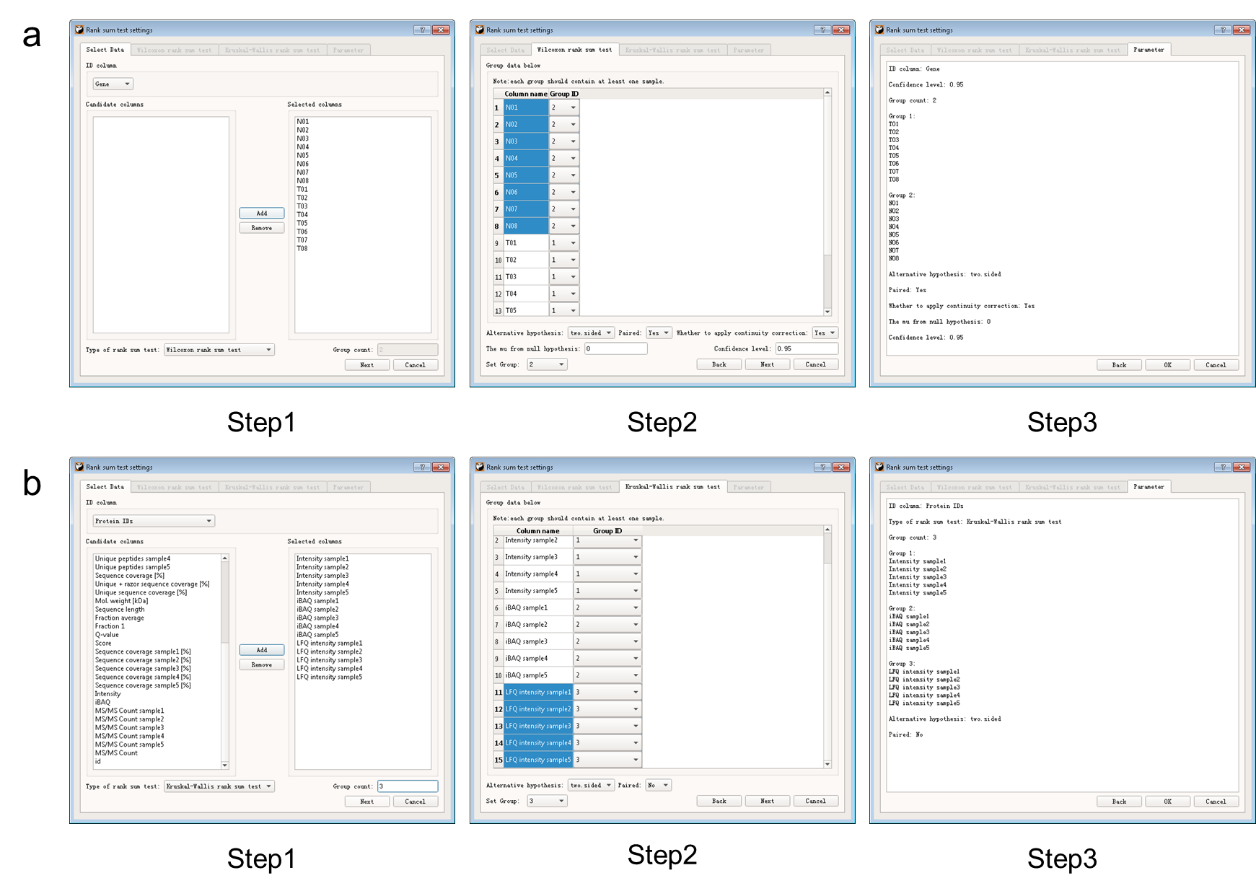


Figure 23. Parameter setting for Wilcoxon rank sum test (a) and Kruskal–Wallis rank sum test (b). Step1: select data and analysis method. Step2: set group ID and parameters. Step3: check the selected data and parameters.


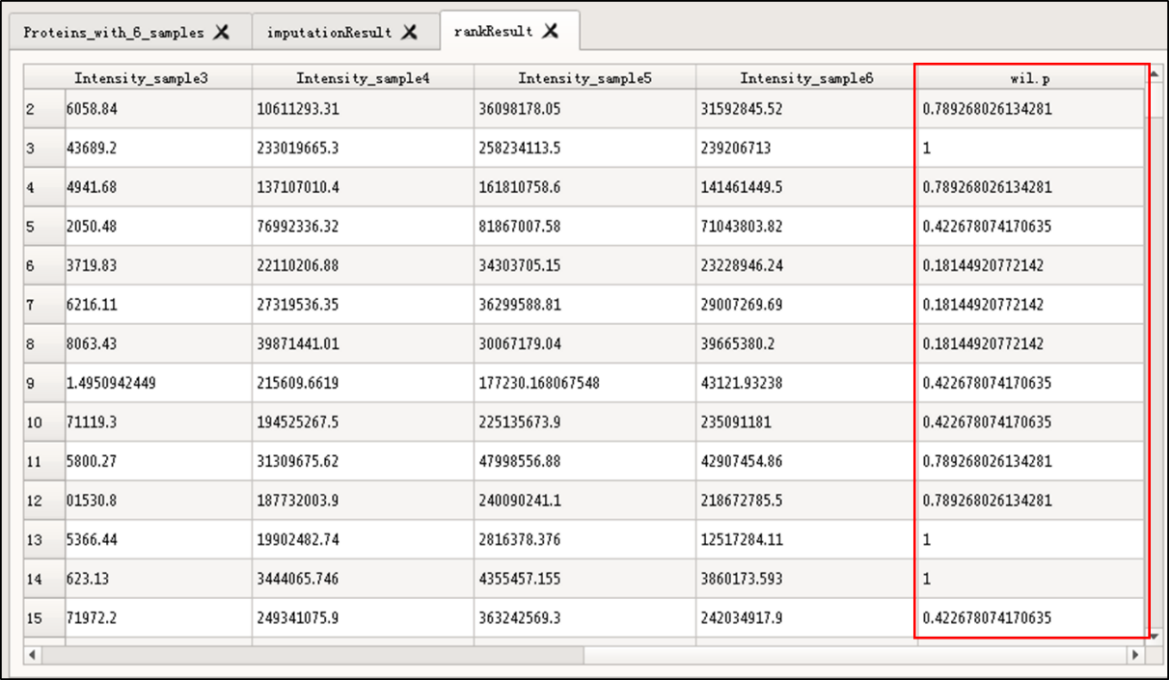


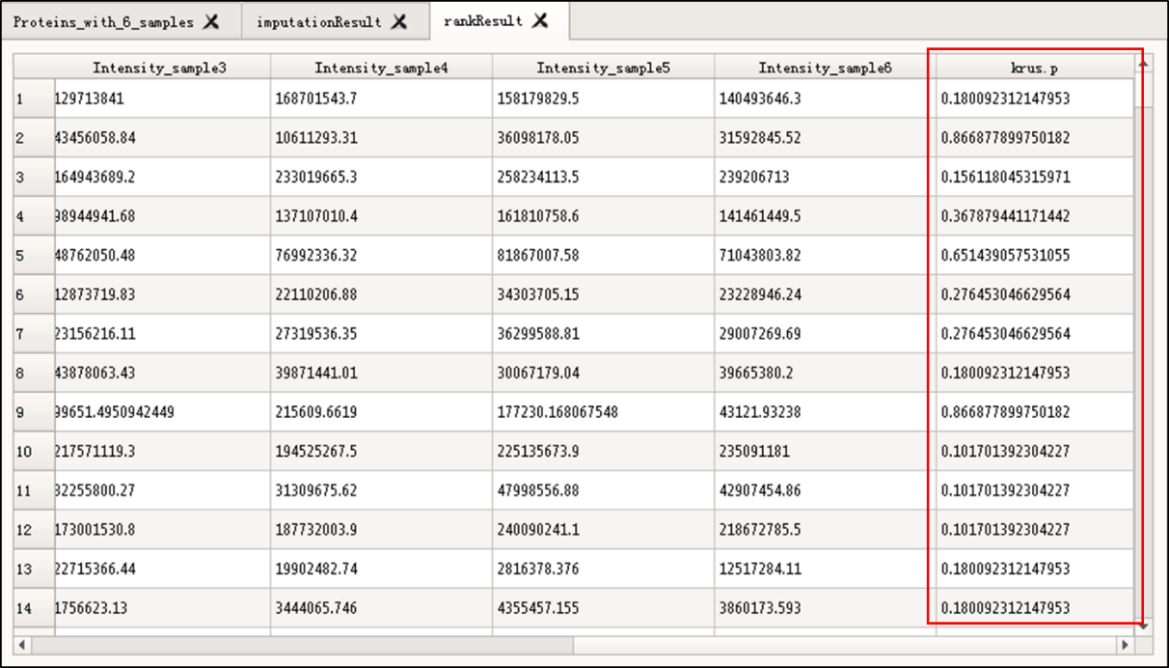


Figure 24. Output examples of Wilcoxon rank sum test and Kruskal–Wallis test.

### 3.3.6 Permutation test

PANDA-view provides *Permutation test* function to judge whether the means of two groups are equal or which is larger. This function is recommended to use in case of insufficient sample size or unknown distribution.

Users can activate the function by clicking the *Permutation test* item in *Statistical Analysis* or the same symbol in *Menu*. The number of samples under each condition is not limited (Fig. 25). The p-value for each row will be given as a new table after calculation (Fig. 26). This method is based on the R packages *splines, survival* and *coin*.


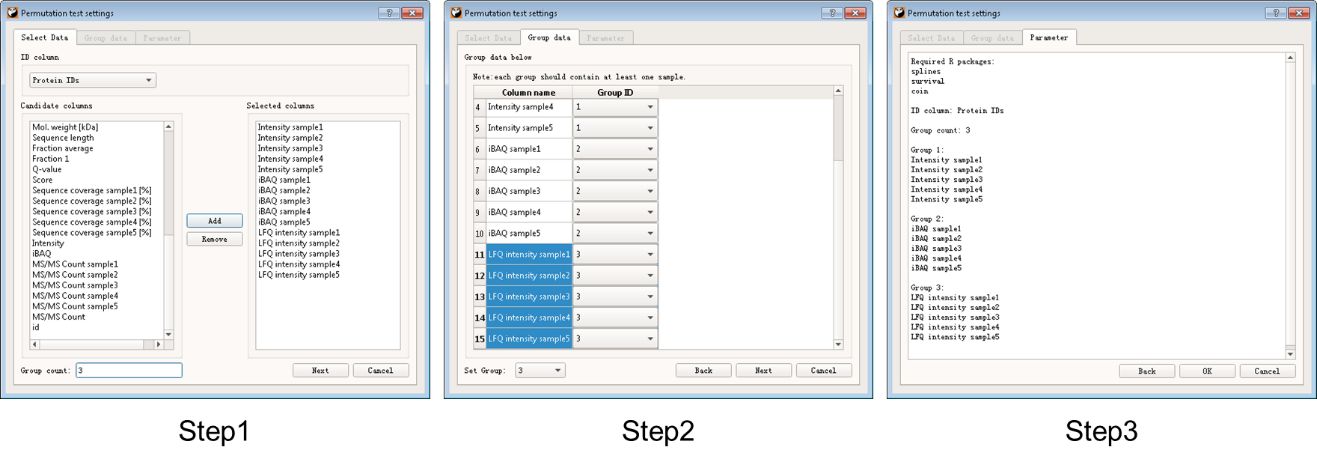


Figure 25. Parameter setting for Permutation test. Step1: select data and set group number. Step2: set group ID and parameters. Step3: check the selected data and parameters.


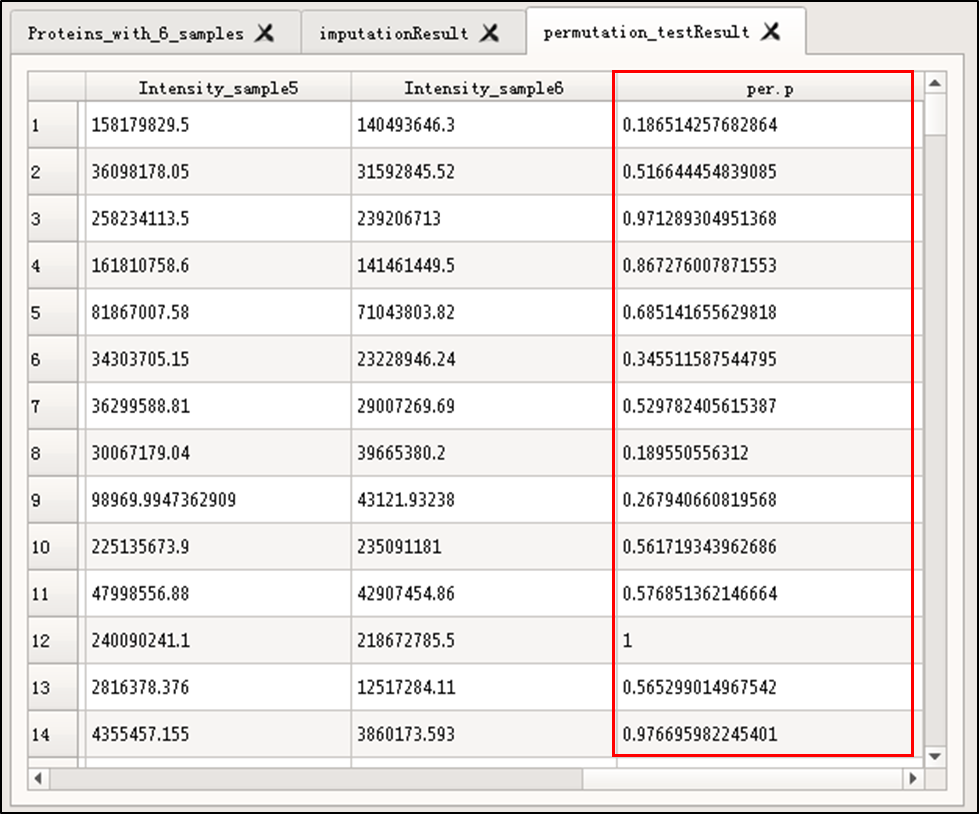


Figure 26. An output example of Permutation test.

### 3.3.7 SAM

Significance analysis of microarrays (SAM) is provided to determine whether the abundance changes of proteins/genes are statistically significant. This analysis uses non-parametric statistics, so the data may be not required to take logarithmic conversion.

By clicking the *SAM* item in *Statistical Analysis* or the same symbol in *Menu*, users can enter the *SAM setting* pop-up window in which *Selected data, Logarithm, Paired* and *Group ID* should be set. After calculation, PANDA-view will output the p-value as a new table for each row (Fig. 27). This method is based on the R packages *R.methodsS3, matrixStats, impute* and *samr*.


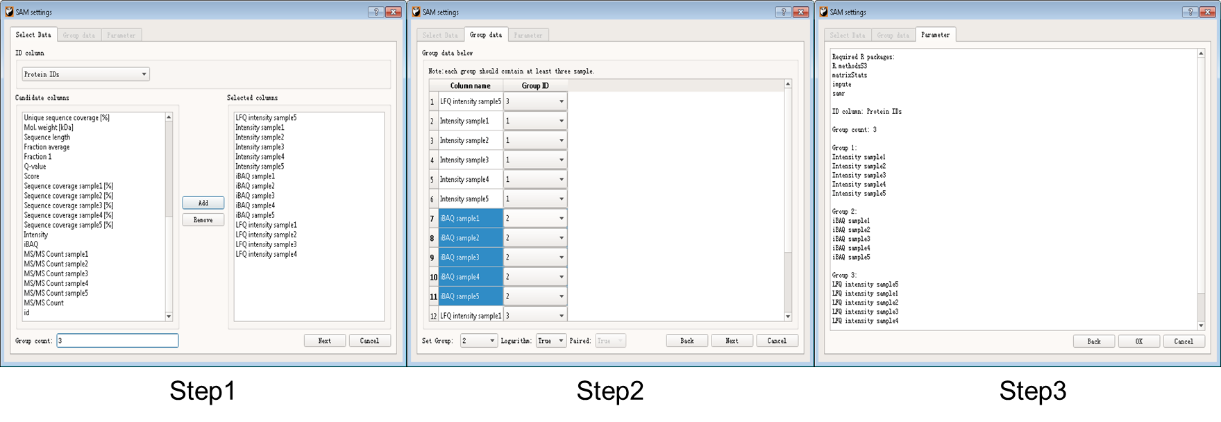


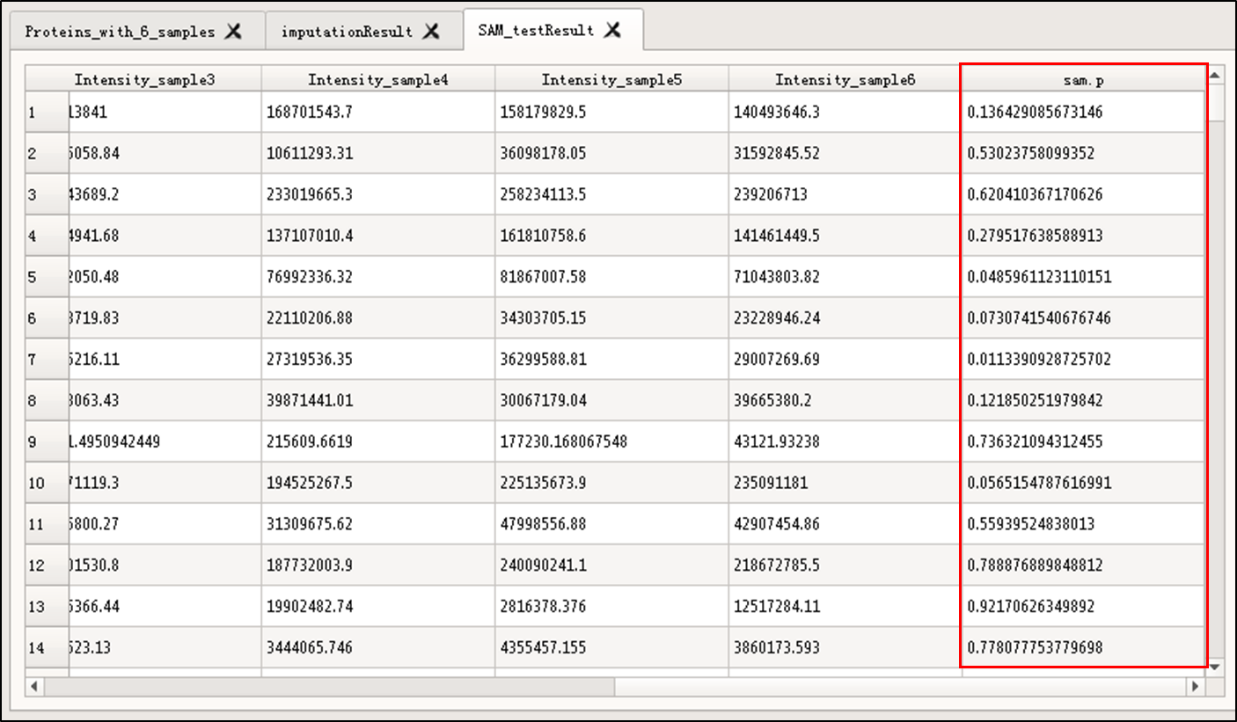


Figure 27. Parameter setting (top) and an example result (bottom) of SAM. Step1: select data and set group number. Step2: set group ID and parameters. Step3: check the selected data and parameters.

### 3.3.8 Multiple hypothesis test

Multiple comparisons, multiplicity or multiple testing problem occurs in biological data analysis frequently. In PANDA-view, several prevalent methods to adjust p-value in multiple hypothesis test are implemented (Fig. 28), such as the Bonferroni method ([Dunn, 1961](#_ENREF_3)), the Benjamini–Hochberg method ([Benjamini and Hochberg, 1995](#_ENREF_1)) and the Benjamini–Yekutieli method ([Benjamini and Yekutieli, 2001](#_ENREF_2)). This method is based on the R function *p.adjust*.


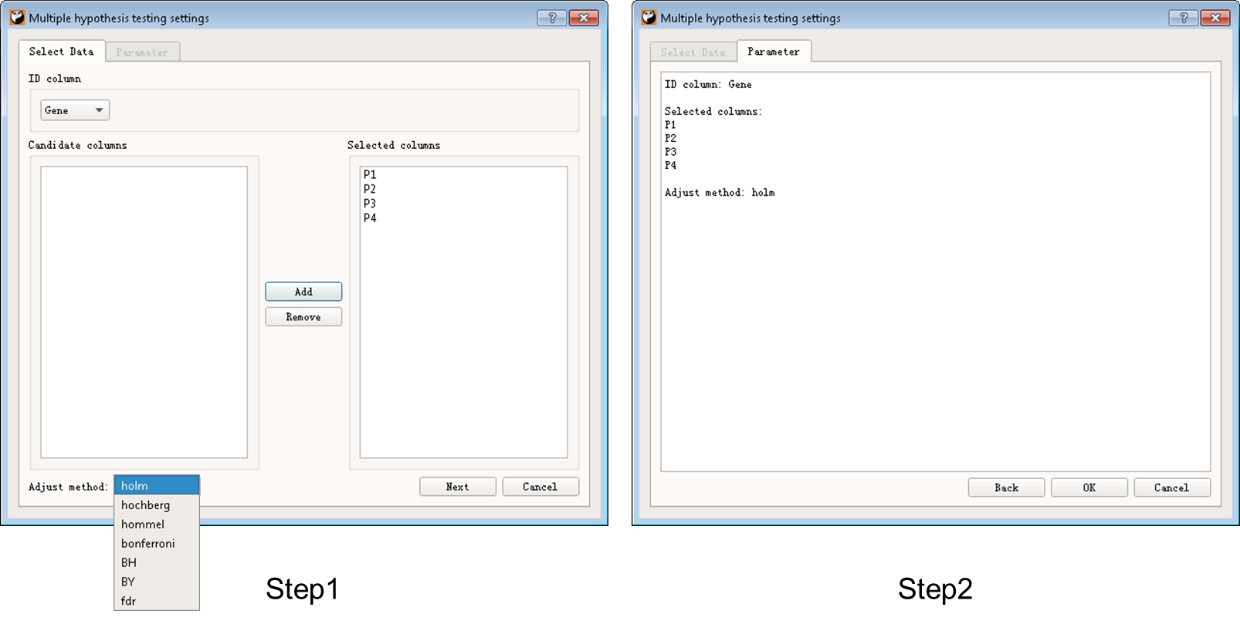


Figure 28. Parameter setting for multiple hypothesis test. Step1: select data and analysis method. Step3: check the selected data and parameters.

## 3.4 Unsupervised Analysis

### 3.4.1 Hierarchical clustering

Hierarchical clustering is often used for a global analysis of -omics data. Here, users can define the clustering methods and distance methods, as well as the parameters of the exported image (Fig. 29). There are two modes for clustering analysis, i.e., simple mode (Fig. 29a) and advanced mode (Fig. 29b). In the advanced mode, nine parameters are added for users to adjust their figures. This method is based on the R function *heatmap2* and the R packages *RColorBrewer* and *gplots*.


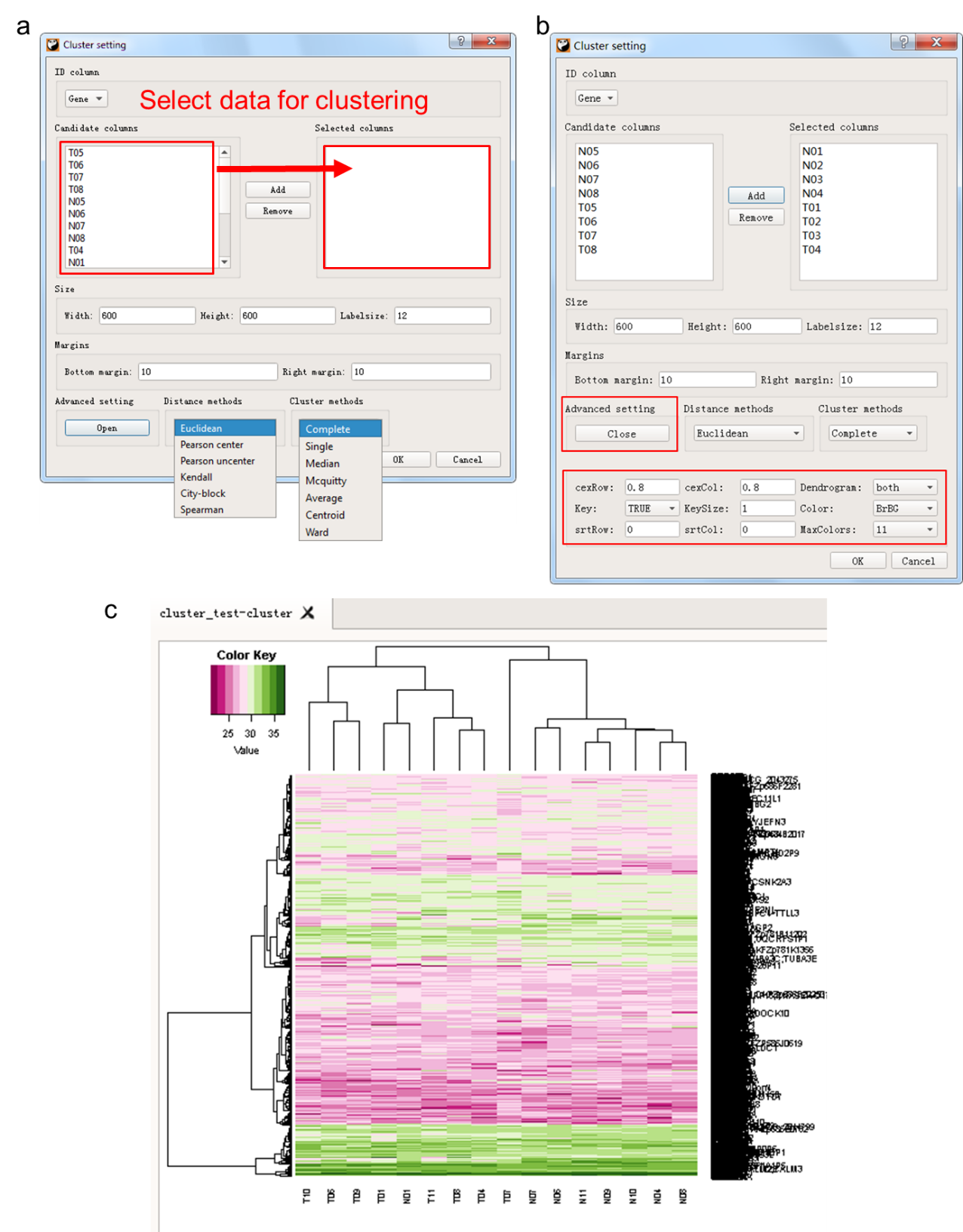


Figure 29. Parameter setting and an example output of hierarchical clustering. (a) simple mode. (b) advanced mode. (c) an example output.

### 3.4.2 K-means clustering

K-means clustering is often used for extremely large data due to its high efficiency compared with hierarchical clustering. Users should set the initial cluster number (K) according to the experiences (Fig. 30). This method is based on the R function *kmeans*.


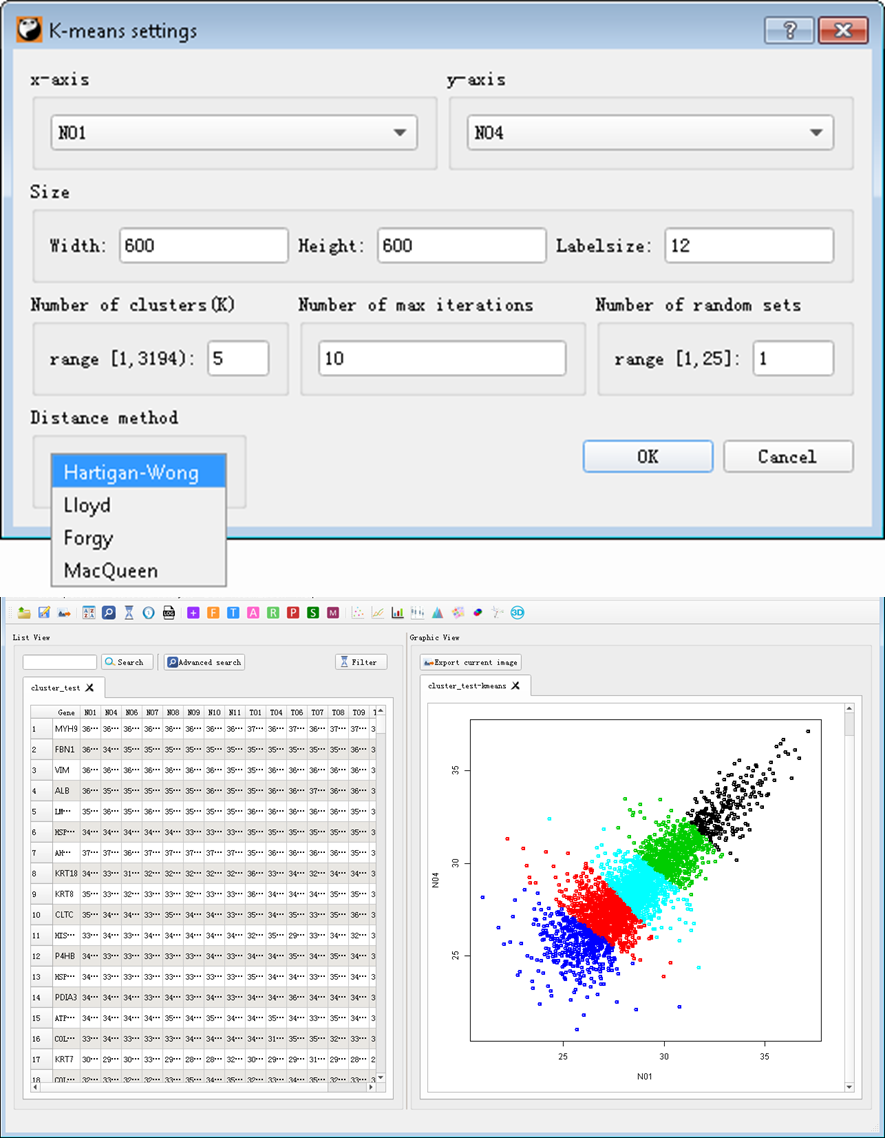


Figure 30. Parameter setting and an example output of K-means clustering.

### 3.4.3 PCA

Principal component analysis (PCA) is a statistical procedure, which is often used to analyze high dimensional -omics data. Here, both 2D and 3D PCA plots are implemented in PANDA-view. As shown in Fig. 31 and Fig. 32, users can perform PCA for their data by simply setting some basic parameters. PCA is based on the R function *princomp*, and 3D PCA plot is additionally based on the R packages *rgl* and *scatterplot3d*.


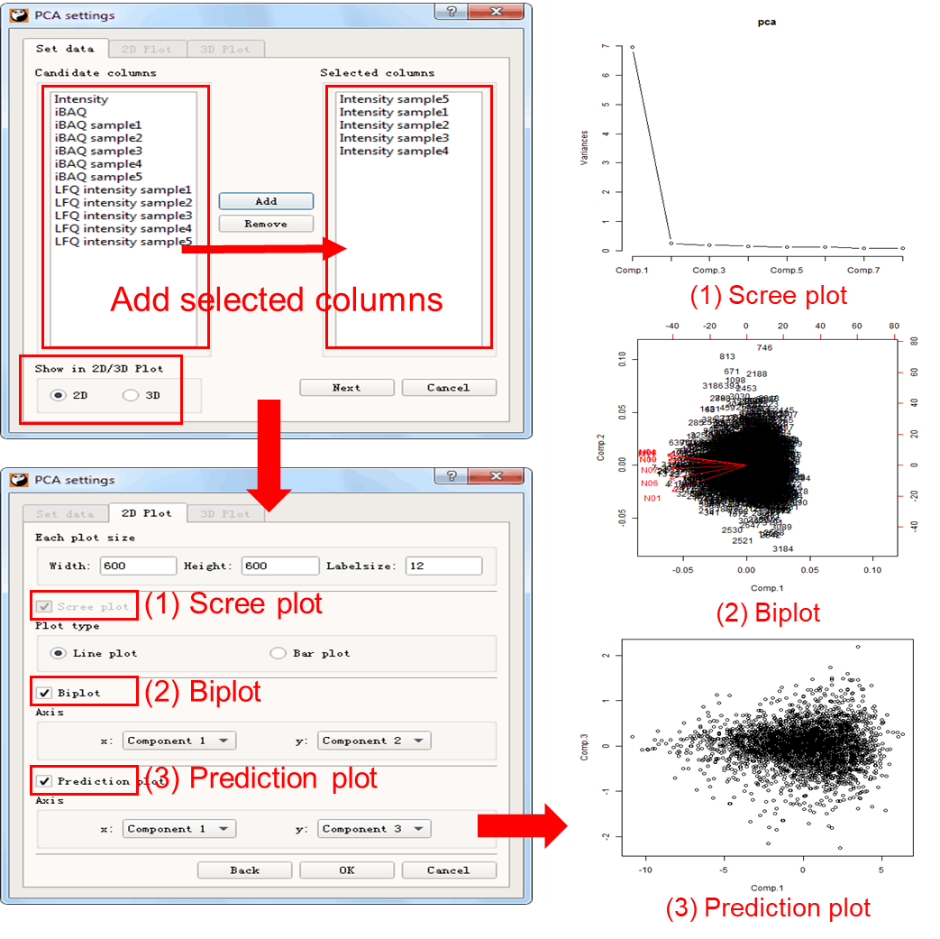


Figure 31. Parameter setting and an example output of PCA in 2D.


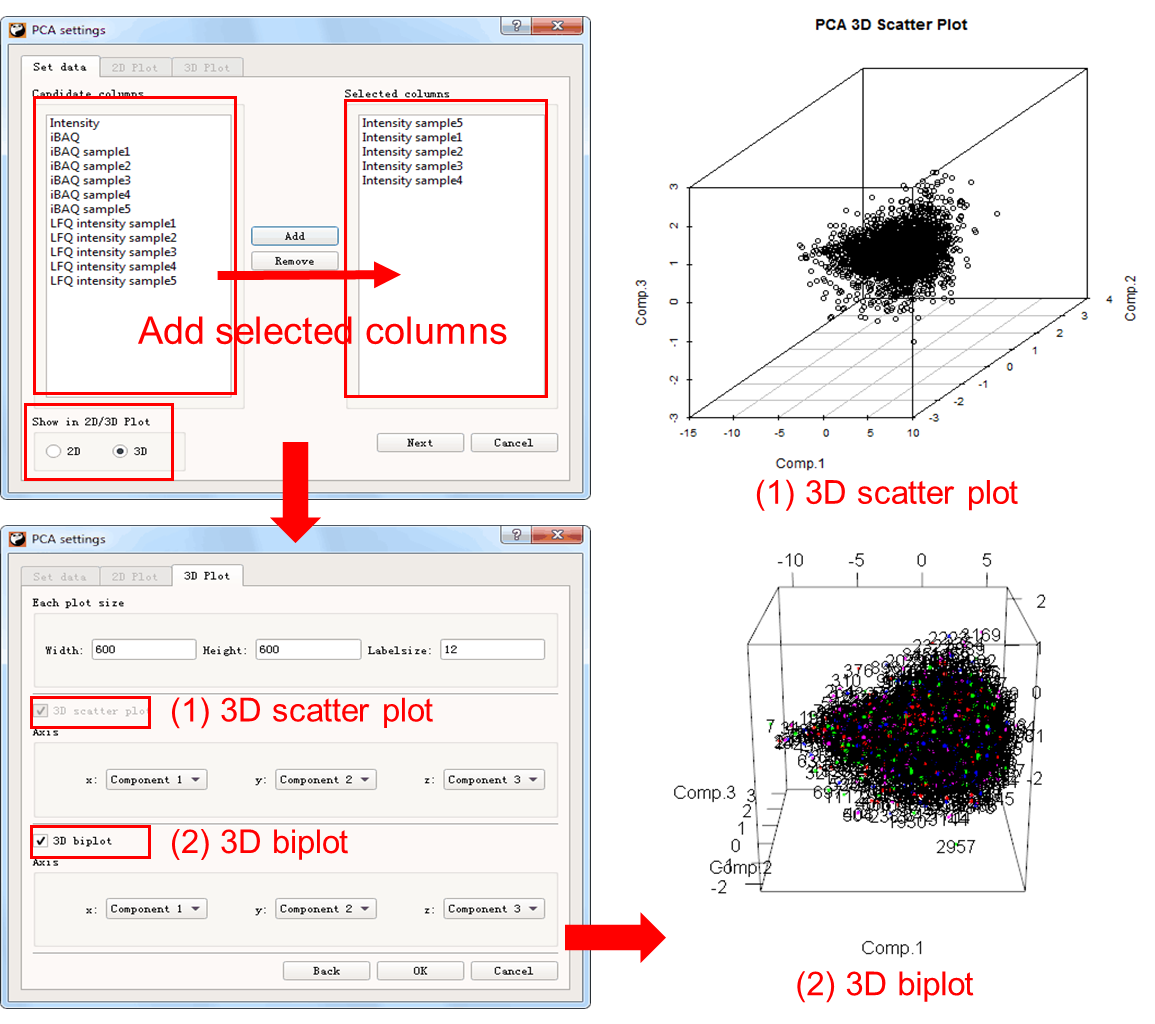


Figure 32. Parameter setting and an example output of PCA in 3D.

## 3.5 Data Visualization

### 3.5.1 2D Scatterplot

Scatter plot is a type of mathematical diagram using Cartesian coordinates to display values for two variables in a dataset. Users should set the axis and decide whether the point can be clicked in PANDA-view. Note that we can click the data point in scatterplot to show its value if we set checkable in PANDA-view (Fig. 33 and Fig. 34).


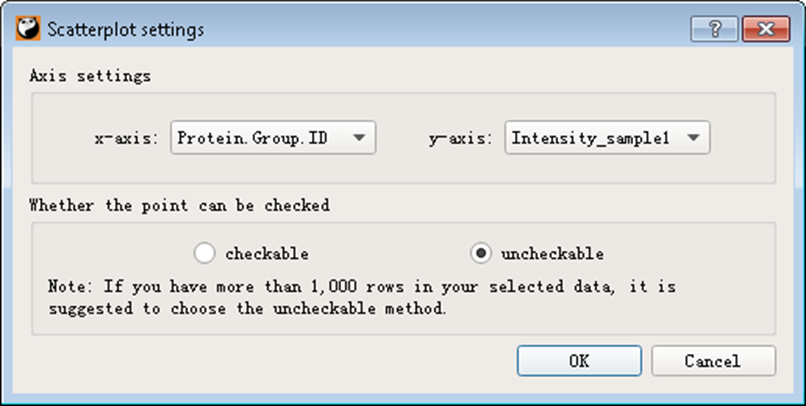


Figure 33. Parameter setting for 2D scatterplot.


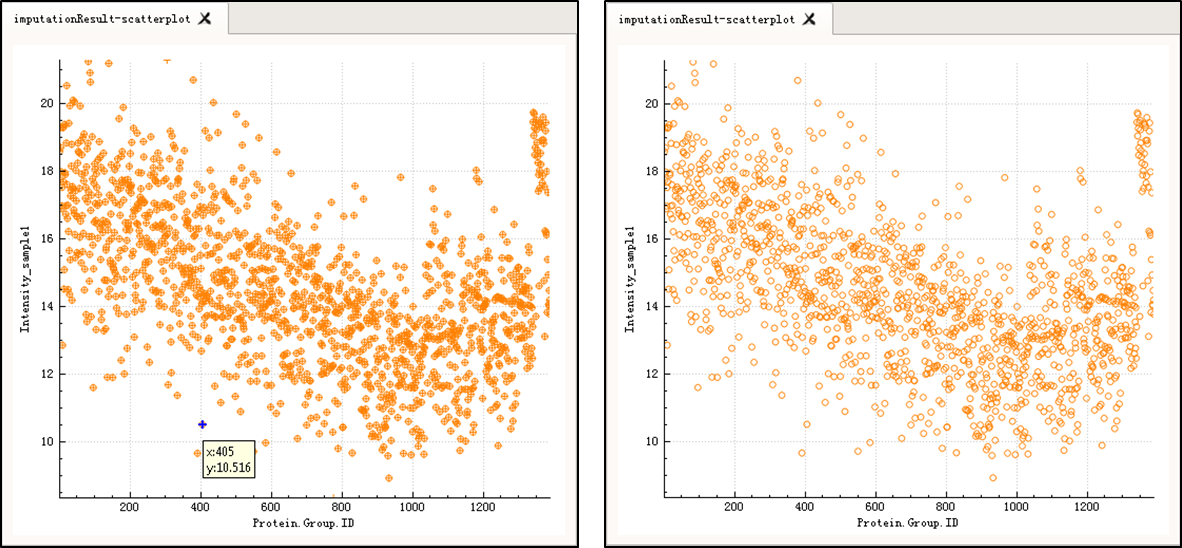


Figure 34. Scatterplots with checked point and unchecked point.

### 3.5.2 3D Scatterplot

Besides 2D scatterplot, PANDA-view also provides a 3D scatterplot function. The x, y and z axes as well as the point size should be set by users at first. The 3D scatterplot can be rotated in 360 degrees and the color of data points changes automatically according to the numerical value (Fig. 35).


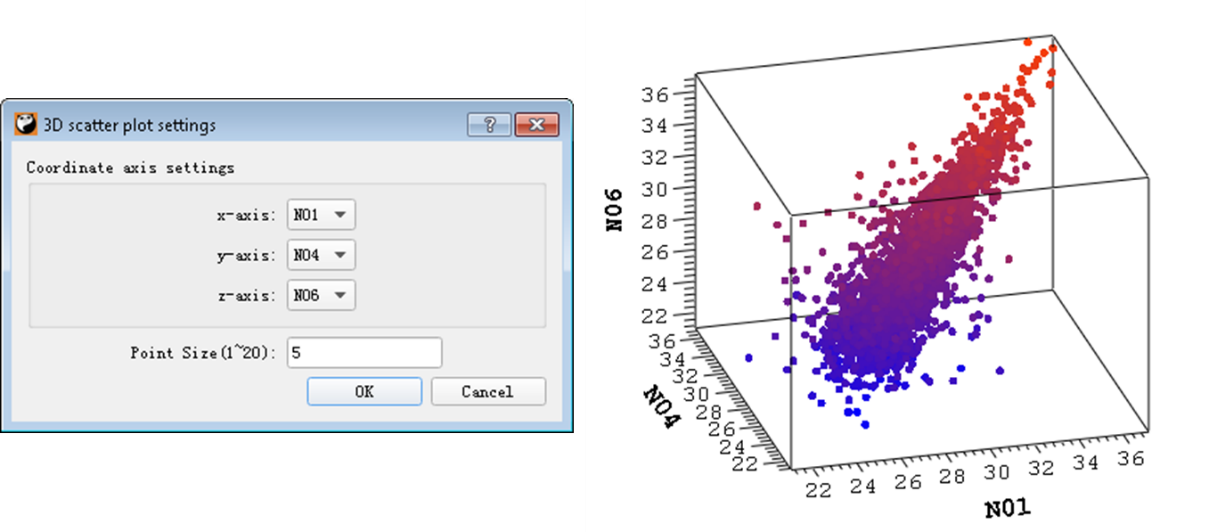


Figure 35. Parameter setting and an example output of 3D scatter plot.

### 3.5.3 Line chart

Line chart is similar to 2D scatter plot except that the measurement points are often ordered (typically by their x-axis value) and joined with straight line segments. Detailed information is shown in Fig. 36 and Fig. 37. Note that every data point in the line chart can be clicked to show its value if users set checkable.


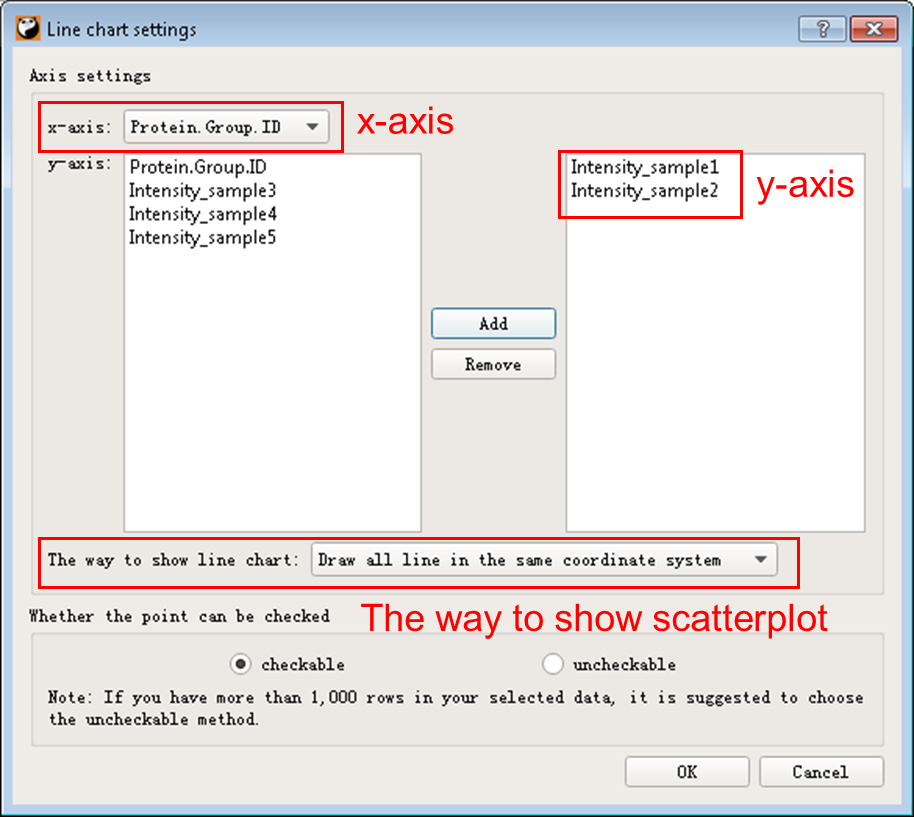


Figure 36. Parameter setting of line chart.


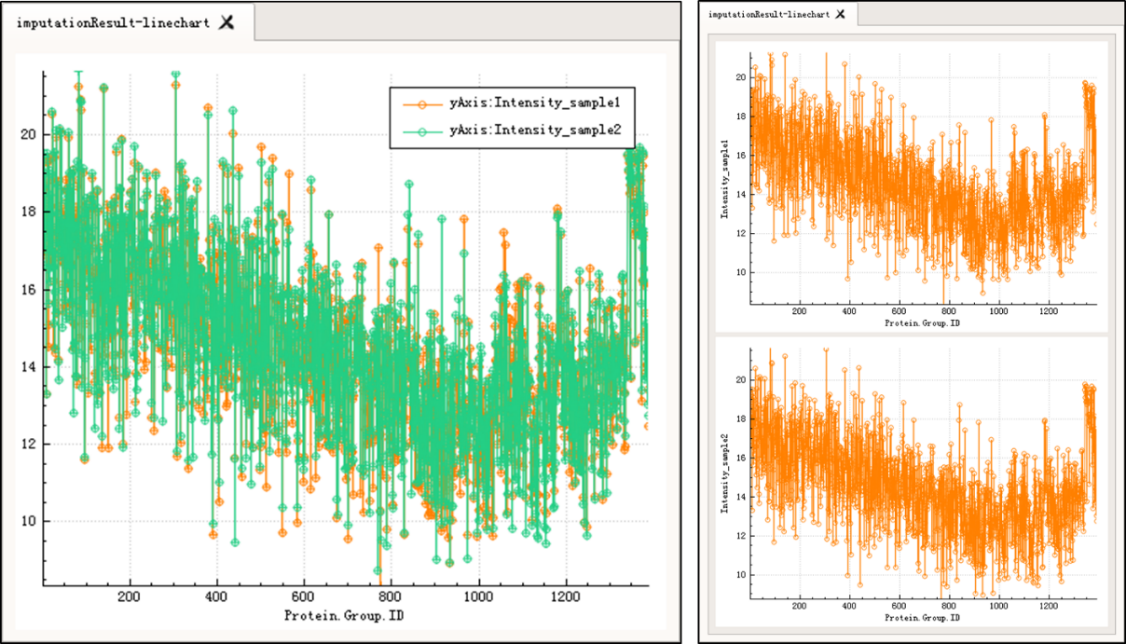


Figure 37. Line chart drawn in the same and different coordinates.

### 3.5.4 Histogram

Histogram is an accurate representation of the distribution of numerical data. It is an estimate of the frequency distribution of a quantitative variable. Users can display count or frequency as they like (Fig. 38).


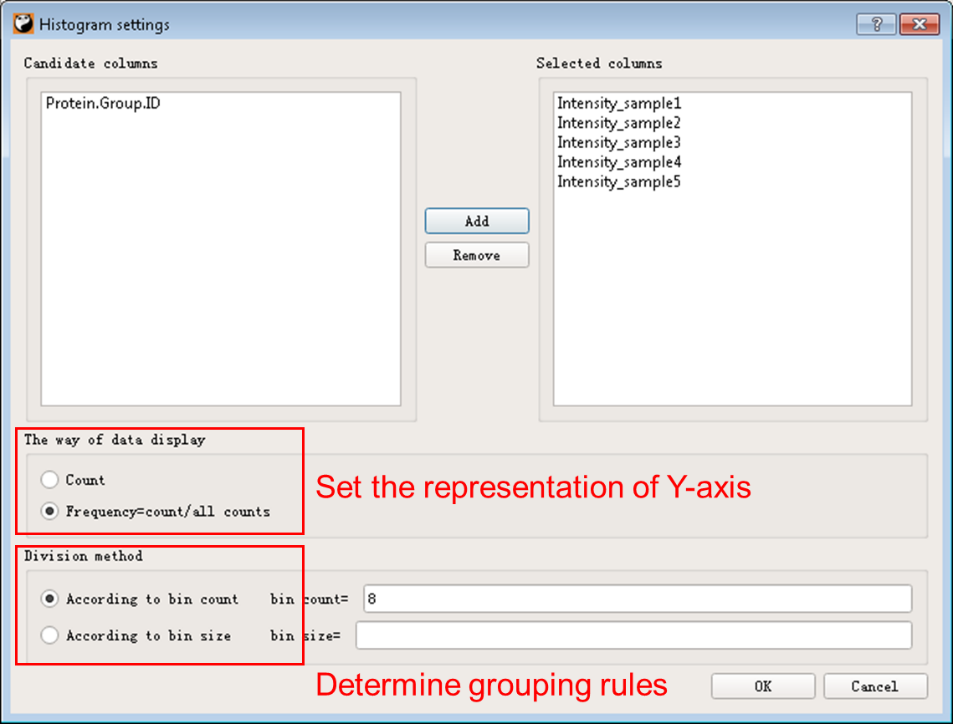


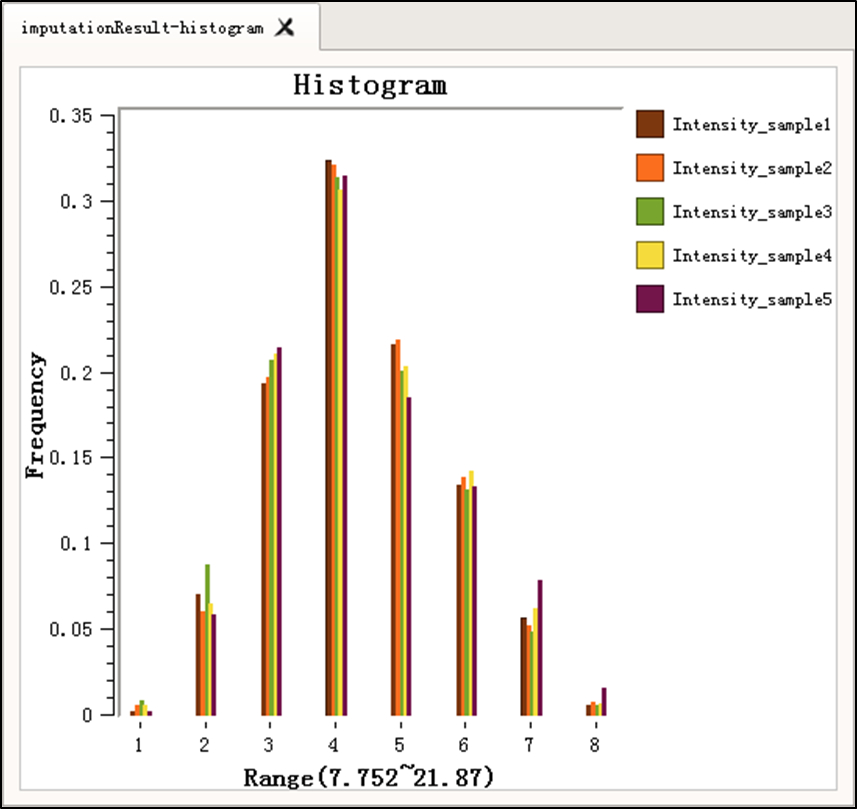


Figure 38. Parameter setting and an example output of histogram.

### 3.5.5 Boxplot

Boxplot is a method for graphically depicting groups of numerical data through their quartiles as well as lines extending vertically from the boxes (whiskers) indicating variability outside the upper and lower quartiles. Detailed information is shown in Fig. 39.


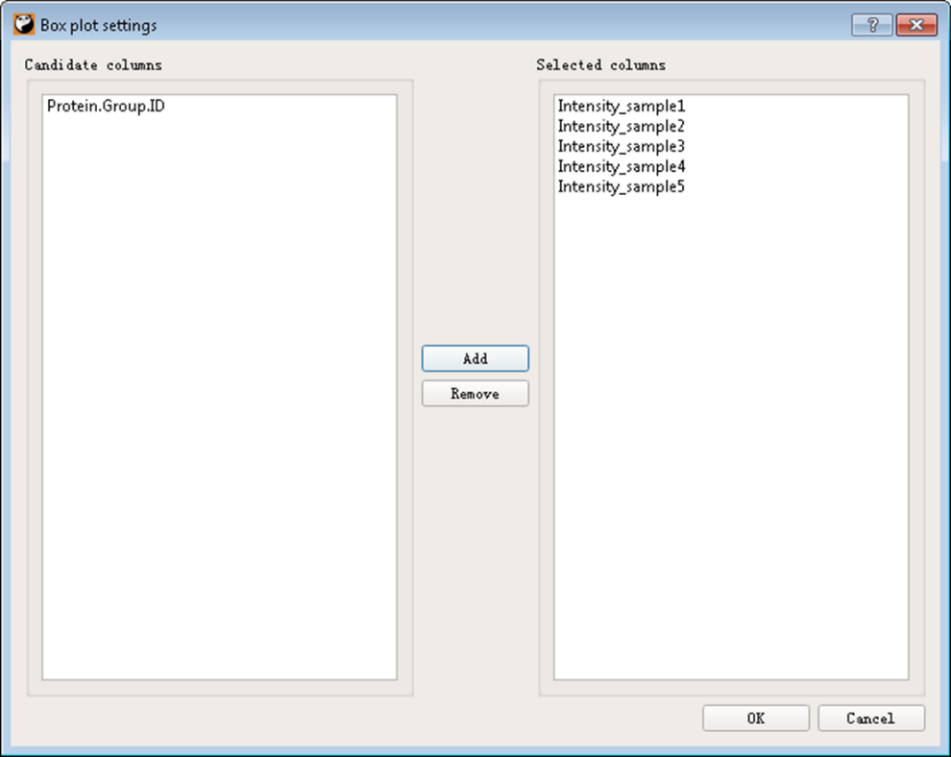


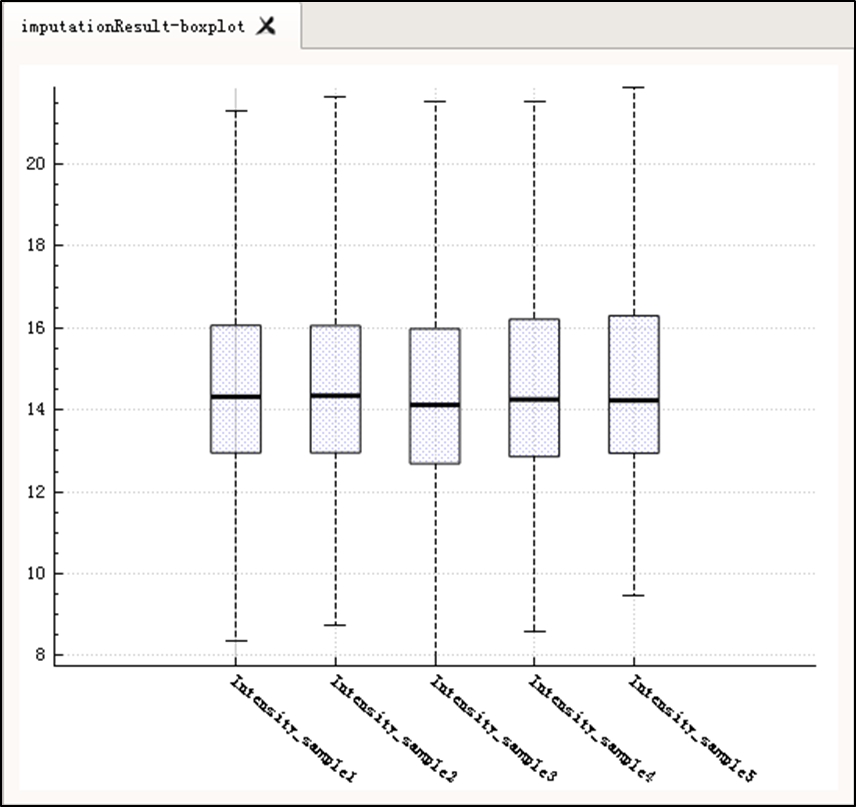


Figure 39. Parameter setting and an example output of boxplot.

### 3.5.6 Volcano plot

Volcano plot is a special kind of scatter plot to show and identify changes in large datasets in biology. Usually, it plots significance (p-value) versus fold-change on the y and x axes, respectively. Volcano plot is often used to show the results of differentially-expressed proteins (DEP) detection in -omics data. The basic parameters are shown in Fig. 40. Further, in PANDA-view, the volcano plot is interactive for users can search the plot using their own key words and the retrieved results will be highlighted in the figure in a different color (Fig. 41). In this function, users need to submit their own table with p-value and ratio.


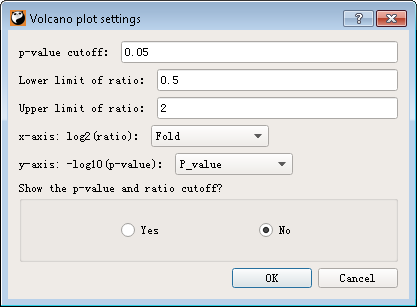


Figure 40. Parameter setting for volcano plot.


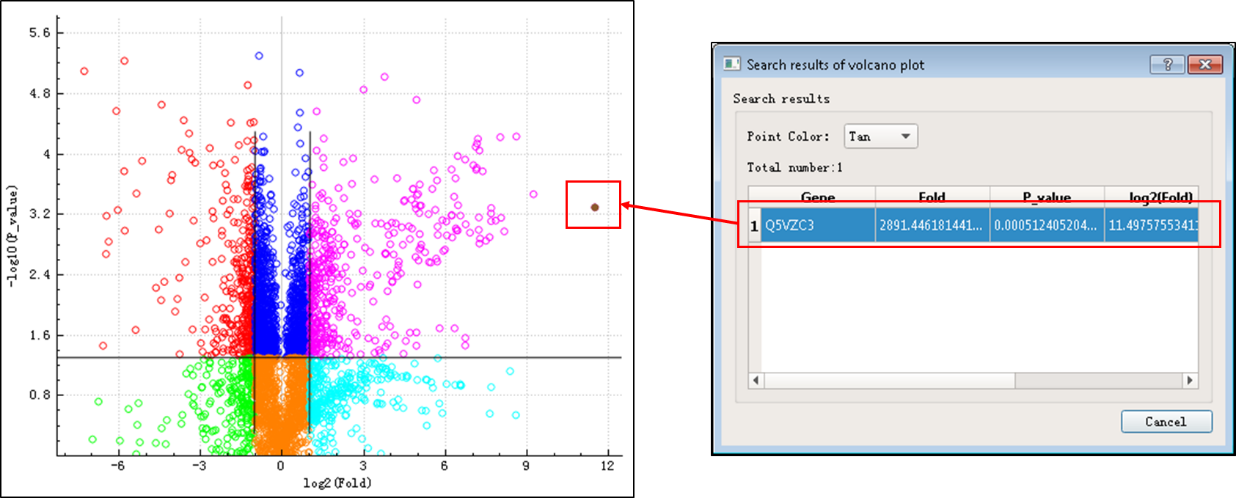


Figure 41. Example of the search function in volcano plot. The searched data will be highlighted in the figure.

## 3.6 Multi-level Representation of Quantitative Data

In addition to the kinds of analysis methods and data visualization methods, PANDA-view can read the quantification results of PANDA directly and perform a multi-level representation of the quantitative data.

PANDA-view can automatically recognize the input files as *Protein QuantResults, Peptide QuantResults* or *PeptideIons QuantResults* when taking PANDA’s results as input (Fig. 42). In case that the key columns were changed, PANDA-view supports the user-defined sub-tables or sub-images based on the index columns. Users can manually select the *File type*, the *Key column of this table* and the *Key columns to index sub-table*. Users can do this by right-clicking on the *Key columns to index sub-table*. Other inputs will be identified as *Other File Types* in which the *Key column of this table* and the *Key columns to index sub-table* are not required to be selected. As shown in Fig. 43, PANDA-view can track a protein to its quantified peptides and then to the corresponding peptide ions with the extracted ion chromatography (XIC) views. Thus, a multi-level view of the proteomic quantification results (protein, peptide, peptide ion and XIC) can be performed in PANDA-view, which is expected to help users make an in-depth analysis of their data.


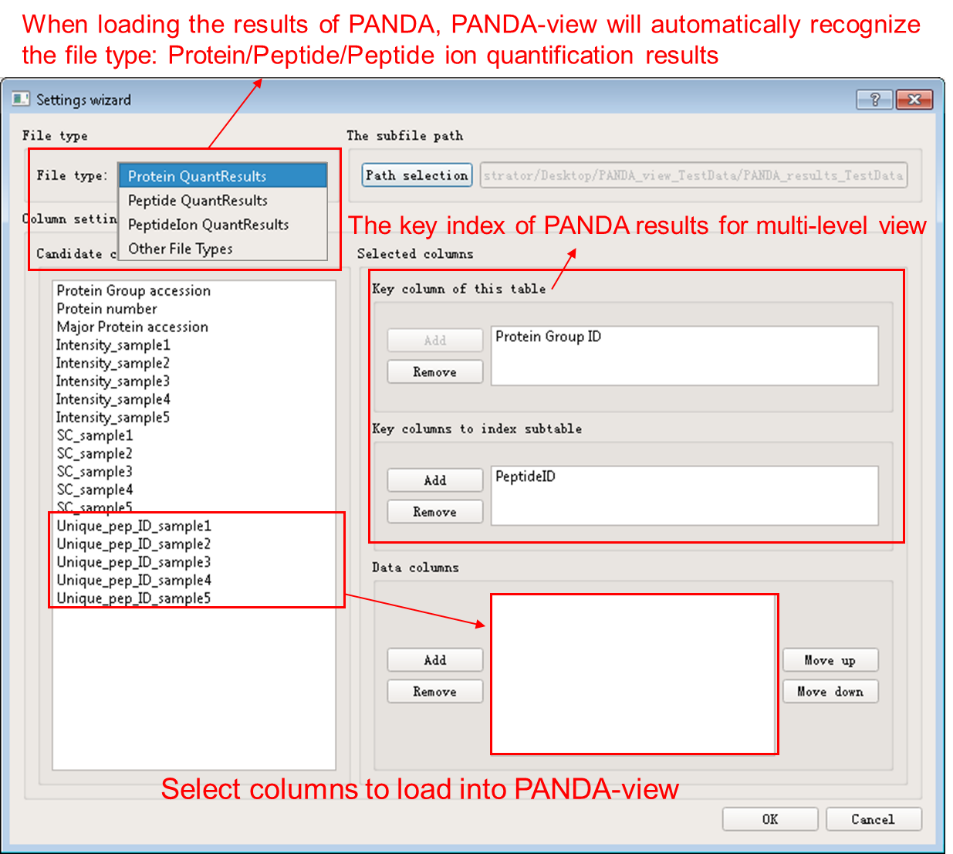


Figure 42. GUI for uploading the results of PANDA.


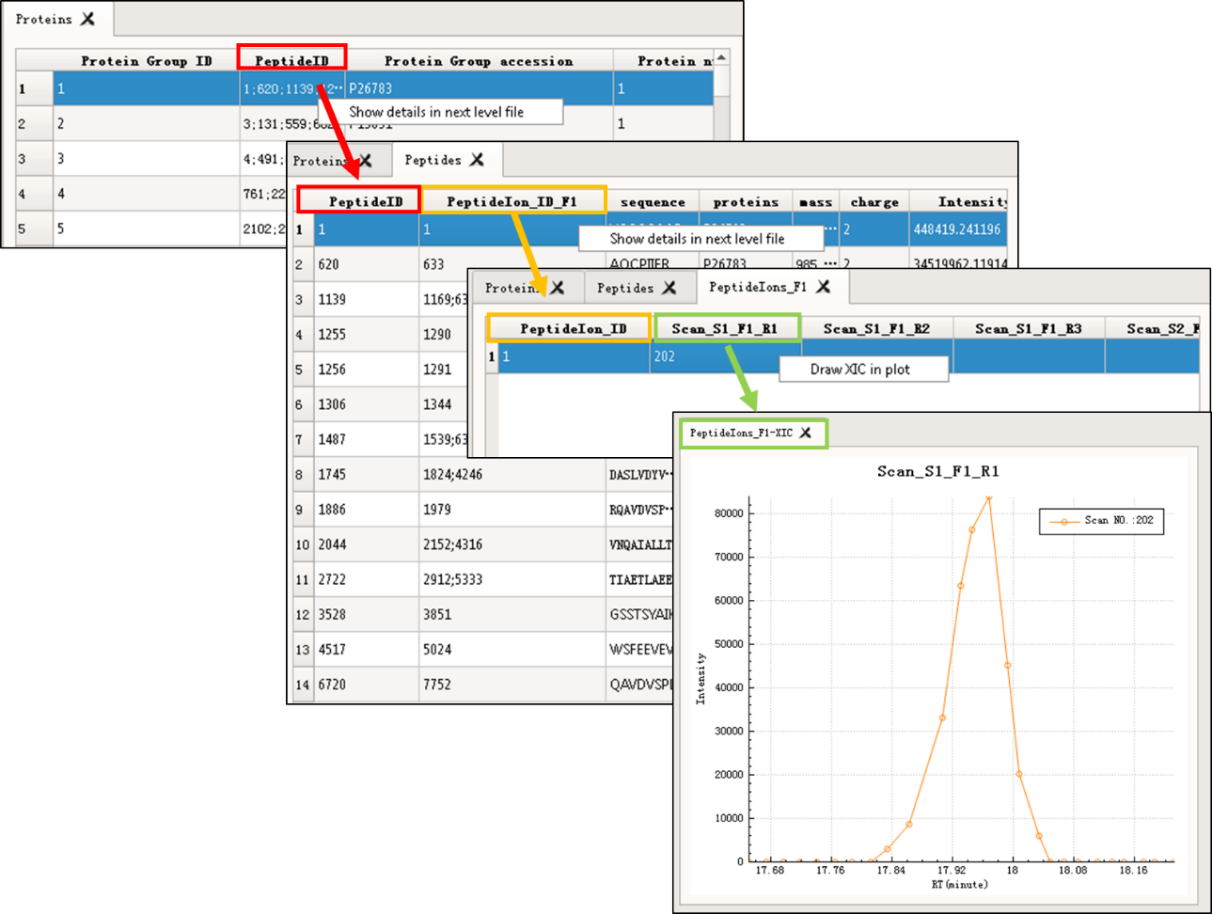


Figure 43. Multi-level representation of quantitative data: protein list 🡪 peptide list 🡪 peptide ion list 🡪 XIC view.

## 3.7 Help

### 3.7.1 Language

PANDA-view supports bilingual display. The software interface can switch between Simplified Chinese and English if users click the *Language* item in *Help* (Fig. 44).


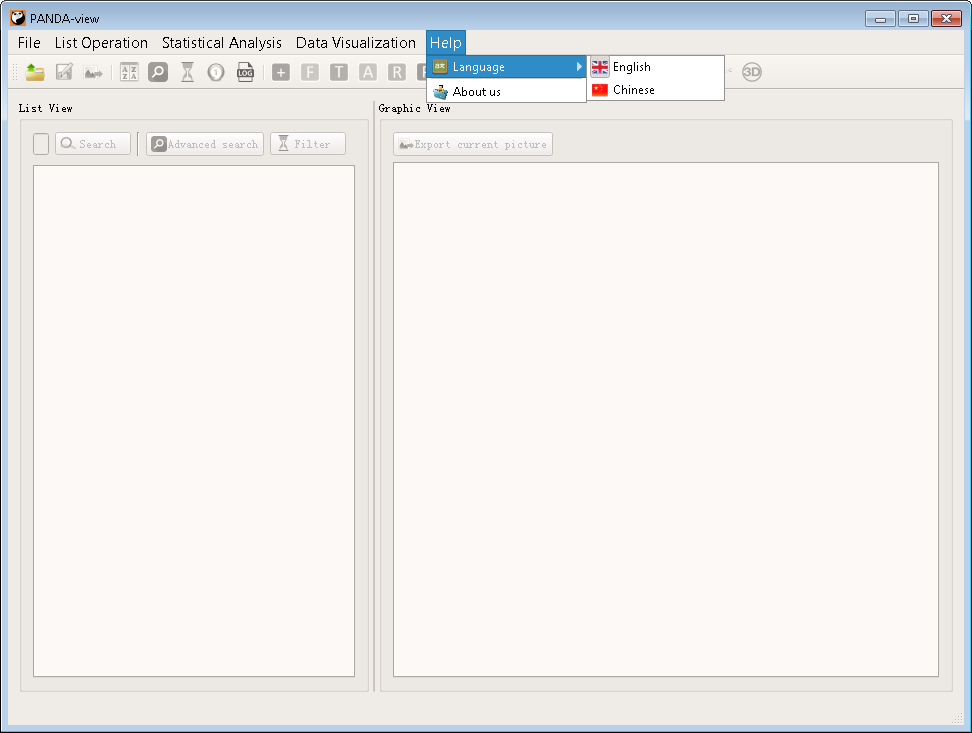


Figure 44. The *Language* item in *Menu*.

### 3.7.2 R requirement

Once users click the “R requirement” button, PANDA-view will automatically check if the path of Rscript.exe is added into the system environment variable and if all the necessary R packages are installed correctly (Fig. 45a). Then, the R version and all the necessary R packages used in PANDA-view will be shown in a pop-up dialog (Fig. 45b). If the system environment variable does not contain the path of Rscript.exe, an error will be popped up (Fig. 46a) and the “R requirement” dialog will be empty (Fig. 46b).


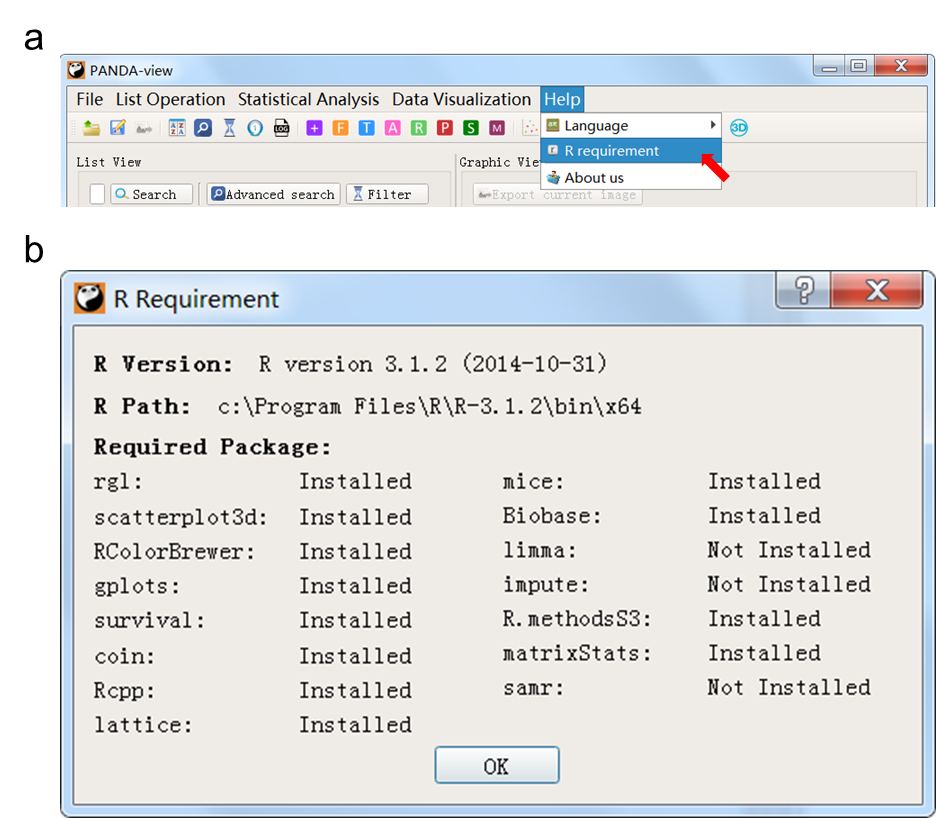


Figure 45. Initial check of R version and the required R packages in PANDA-view. (a) the “R requirement” button in the menu to check R version and the required R packages. (b) the pop-up dialog showing the R version used in PANDA-view and the installation status of each necessary R package. Users should re-install the packages whose statuses are “Not Installed”.


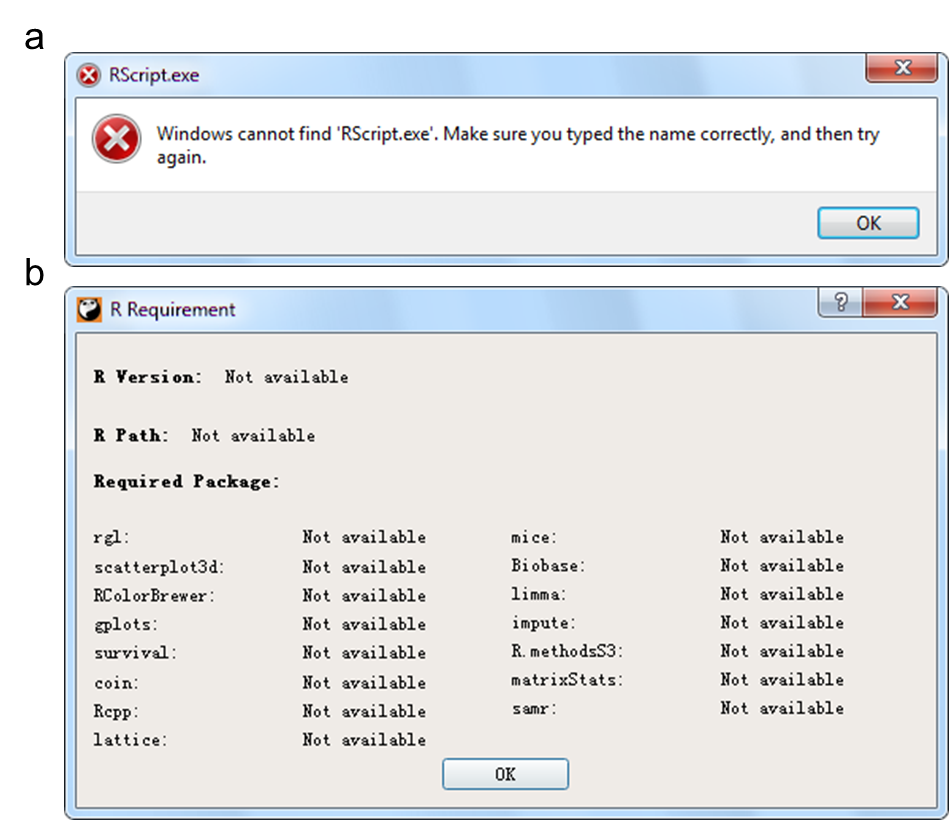


Figure 46. The situation when the system environment variable does not contain the path of Rscript.exe. (a)The error dialog. (b) The empty dialog about R version and R packages.

### 3.7.3 About us

Some detailed information about the developers will be presented to users once the *About us* item is clicked. (Fig. 47).


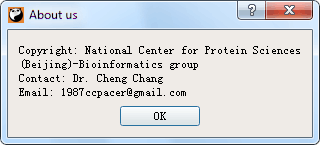


Figure 47. The *About us* item in *Menu*.

# Chapter 4. Support Services

## 4.1 Contact

For any questions involving PANDA-view, please contact Dr. Cheng Chang (Email: [1987ccpacer@163.com](mailto:1987ccpacer@163.com) or [1987ccpacer@gmail.com](mailto:1987ccpacer@gmail.com)).

## 4.2 Copyright

This software product is developed by Dr. Cheng Chang from the National Center for Protein Sciences (Beijing)-Bioinformatics group. All titles and intellectual property rights, which is generated by the software product including, but not limited to, relative images, data, texts, additional program and other software products (dll, exe, etc.), incidental help materials, and any copies of the Software Products are protected by Copyright Law of People’s Republic of China and international copyright treaties and other intellectual property laws and treaties. Users only get the right to use this software product for non-commercial uses.

# Chapter 5. References

Benjamini, Y. and Hochberg, Y. (1995) Controlling the False Discovery Rate: A Practical and Powerful Approach to Multiple Testing, *Journal of the Royal Statistical Society. Series B (Methodological)*, **57**, 289-300.

Benjamini, Y. and Yekutieli, D. (2001) The control of the false discovery rate in multiple testing under dependency, *Ann. Statist.*, **29**, 1165-1188.

Dunn, O.J. (1961) Multiple Comparisons among Means, *Journal of the American Statistical Association*, **56**, 52-64.

Huber, W.*, et al.* (2002) Variance stabilization applied to microarray data calibration and to the quantification of differential expression, *Bioinformatics*, **18 Suppl 1**, S96-104.

Ritchie, M.E.*, et al.* (2015) limma powers differential expression analyses for RNA-sequencing and microarray studies, *Nucleic Acids Res*, **43**, e47.

Valikangas, T., Suomi, T. and Elo, L.L. (2018) A systematic evaluation of normalization methods in quantitative label-free proteomics, *Brief Bioinform*, **19**, 1-11.
